# Exploratory Analysis of Coagulation and Fibrinolysis Trajectories After IL-6 Antagonist Therapy in COVID-19: A Case Series

**DOI:** 10.3390/biomedicines14010254

**Published:** 2026-01-22

**Authors:** Emőke Henrietta Kovács, Máté Rottler, Zoltán Ruszkai, Csanád Geréd, Tamás Kiss, Margit Csata, Barbara Réger, Rita Jakabfi-Csepregi, István Papp, Caner Turan, Péter Hegyi, János Fazakas, Zsolt Molnár, Krisztián Tánczos

**Affiliations:** 1 Centre for Translational Medicine, Semmelweis University, 1085 Budapest, Hungary; kovacs.emoke@phd.semmelweis.hu (E.H.K.); hegyi.peter@semmelweis.hu (P.H.); zsoltmolna@gmail.com (Z.M.); 2 Department of Anaesthesiology and Intensive Therapy, Szent György University Teaching Hospital of Fejér County, 8000 Székesfehérvár, Hungary; 3 Doctoral School of Clinical Medicine, University of Szeged, 6720 Szeged, Hungary; 4 Department of Anaesthesiology and Intensive Therapy, Flór Ferenc County Hospital, 2143 Kistarcsa, Hungary; dr.ruszkai.zoltan@gmail.com (Z.R.); gered.csanad@florhosp.hu (C.G.); 5 Department of Anaesthesiology and Intensive Therapy, University of Pécs, 7624 Pécs, Hungary; kiss.tamas@pte.hu (T.K.); csata.margit@pte.hu (M.C.); 6 Department of Laboratory Medicine, Medical School, University of Pécs, 7624 Pécs, Hungary; reger.barbara@pte.hu (B.R.); papp.istvan@pte.hu (I.P.); 7 Department of Anaesthesiology and Intensive Therapy, Semmelweis University, 1082 Budapest, Hungary; fazakas.janos@semmelweis.hu; 8 Institute for Translational Medicine, Szentágothai Research Centre, Medical School, University of Pécs, 7624 Pécs, Hungary; 9 Institute of Pancreatic Diseases, Semmelweis University, 1083 Budapest, Hungary; 10 Department of Anaesthesiology and Intensive Therapy, Poznan University of Medical Sciences, 60-806 Poznan, Poland

**Keywords:** coagulation, fibrinolysis, COVID-19, thromboinflammation, interleukin-6 antagonist, endothelial dysfunction, viscoelastic hemostatic assay

## Abstract

**Background/Objectives**: Severe COVID-19 is marked by IL-6-driven inflammation, endothelial injury, and dysregulated coagulation. Although IL-6 antagonists improve clinical outcomes, their effects on the temporal evolution of coagulation and fibrinolysis remain insufficiently defined. This study characterizes inflammatory, endothelial, coagulation, and fibrinolytic trajectories following IL-6 receptor blockade in critically ill COVID-19 patients. **Methods**: In this prospective, exploratory multicenter case series (ClinicalTrials.gov NCT05218369), 15 ICU patients with PCR- or antigen-confirmed COVID-19 received tocilizumab per protocol. Serial sampling at five timepoints (T0–T4) included routine laboratories, comprehensive viscoelastic hemostatic assays (ClotPro^®^), and ELISA-based endothelial and fibrinolytic biomarkers. Analyses were primarily descriptive, emphasizing temporal patterns through boxplots; paired Wilcoxon tests with FDR correction contextualized within-patient changes. **Results**: Patients exhibited marked inflammation, hyperfibrinogenemia, endothelial activation, and delayed fibrinolysis at baseline. IL-6 blockade induced rapid suppression of CRP and PCT, progressive declines in fibrinogen, and modest platelet increases. In contrast, vWF antigen and activity further increased, indicating persistent endothelial dysfunction. Viscoelastic testing showed preserved thrombin generation and sustained high clot firmness, while biochemical markers (rising PAI-1, modest PAP increase, and progressively increasing D-dimer) and VHA indices suggested ongoing antifibrinolytic activity despite resolution of systemic inflammation. **Conclusions**: IL-6 antagonism was associated with rapid attenuation of systemic inflammation but was not accompanied by normalization of endothelial activation or fibrinolytic resistance. The observed hemostatic profile was consistent with attenuation of inflammation-associated coagulation features, while endothelial and prothrombotic alterations appeared to persist during follow-up, warranting further investigation in larger controlled studies.

## 1. Introduction

The COVID-19 pandemic unveiled the critical crosstalk between inflammation and coagulation, with cytokine-driven immune activation contributing to a markedly prothrombotic state [1]. The inflammatory and coagulation systems have evolved in close interdependence, a relationship evident throughout phylogeny [2]. Inflammatory stimuli and endothelial injury create a pro-coagulant environment, commonly referred to as thromboinflammation, which not only promotes clot formation but also aids in pathogen containment and immune regulation [3].

Cytokines such as interleukin-6 (IL-6) play a central role in this crosstalk. IL-6 enhances coagulation through multiple mechanisms: upregulating the hepatic synthesis of fibrinogen, increasing expression of factor VIII and tissue factor, and downregulating natural anticoagulants such as antithrombin and protein S. Additionally, IL-6 promotes antifibrinolytic activity by stimulating endothelial cells to produce plasminogen activator inhibitor-1 (PAI-1) via trans-signaling pathways. The cumulative effect of elevated IL-6 is a shift in hemostatic balance toward a procoagulant, hypofibrinolytic state [4].

The abovementioned phenomena are not confined to a single disease. In chronic inflammatory disorders such as rheumatoid arthritis and systemic juvenile idiopathic arthritis, persistently elevated IL-6 contributes to a hypercoagulable state, mirroring the hemostatic disturbances seen in acute inflammation, albeit typically less pronounced [5,6]. Clinical experience and mechanistic studies support the notion that IL-6 is a key driver of inflammation-induced coagulation abnormalities and that IL-6 blockade may reduce thrombotic risk [7].

The prothrombotic effects of IL-6 have also been implicated in systemic inflammatory response (SIRS), where elevated IL-6 levels correlate with increased tissue factor and PAI-1 expression, potentially contributing to disseminated intravascular coagulation (DIC) and multiorgan failure. Interrupting the IL-6/sIL-6R pathway may, therefore, attenuate coagulopathy and improve outcomes [8].

While tocilizumab improves outcomes in severe COVID-19, its influence on the temporal interplay between inflammation, coagulation, and fibrinolysis remains unclear. Given that severe COVID-19 is characterized by both cytokine storm and coagulopathy, we hypothesize that immunomodulation with IL-6 antagonists could mitigate hemostatic disturbances without the need for intensified anticoagulation beyond standard of care [9]. This study characterizes hemostatic alterations in critically ill COVID-19 patients undergoing IL-6 receptor blockade. We assessed longitudinal changes in coagulation and fibrinolytic parameters before and after treatment using ClotPro^®^ viscoelastic assays. We further explored associations between coagulation abnormalities, endothelial dysfunction, and systemic inflammation in this observational setting.

## 2. Materials and Methods

### 2.1. Study Design and Setting

We conducted a prospective, exploratory, multicenter case series involving three Hungarian intensive care units (ICUs). This study complied with the Declaration of Helsinki and was approved by the Hungarian Medical Research Council (1405-3/2022/EÜG). The protocol has been published previously and was registered on ClinicalTrials.gov (NCT05218369) [10].

### 2.2. Eligibility

Patients were enrolled without active recruitment based on the treating physician’s decision to initiate IL-6 receptor blockade according to local institutional protocols.

Inclusion criteria comprised adults with polymerase chain reaction (PCR) or antigen-confirmed SARS-CoV-2 infection and acute respiratory failure requiring immunomodulation. Typical indications included invasive or non-invasive ventilation, or high-flow nasal oxygen therapy (FiO_2_ > 0.4, flow > 30 L/min), in the presence of systemic inflammation defined by C-reactive protein (CRP) > 75 mg/L.

Exclusion criteria included prior administration of immunomodulatory agents, chronic immunosuppression, suspected active bacterial infection, platelet count < 50 × 10^9^/L, recent fibrinolytic therapy or blood product administration, pregnancy, inability to obtain informed consent from the patient or legally authorized representative, and a delay exceeding 120 h between ICU admission and IL-6 antagonist administration.

### 2.3. Data Collection

Prospective data included demographics, comorbidities, clinical status, therapies, blood cultures, routine labs, and viscoelastic hemostatic assays (VHA). Sites entered data into standardized eCRFs in a centralized database; patients were de-identified with unique study IDs.

Blood was sampled prior to tocilizumab administration (T0) and subsequently at 24 h (T1), 48 h (T2), 120 h (T3), and 168 h (T4). Viscoelastic hemostatic assays (VHA; ClotPro^®^, Haemonetics Corporation, Boston, MA, USA) included extrinsic pathway (EX), intrinsic pathway (IN), fibrinogen (FIB), tissue plasminogen activator-enhanced (TPA), Russell viper venom (RVV), and ecarin clotting (ECA) assays. Parameters recorded reflected clot initiation (clotting time, CT), clot propagation and mechanical strength (clot formation time, CFT; maximum clot firmness, MCF), and fibrinolytic activity (maximum lysis, ML; clot lysis index at 30 and 45 min, CLI-30/45; lysis onset time, LOT; and lysis time, LT). Interpretation of the ClotPro assays and derived parameters is summarized in Supplementary Table S4.

Routine laboratory tests, including complete blood count (CBC), C-reactive protein (CRP), procalcitonin (PCT), ferritin, international normalized ratio (INR), activated partial thromboplastin time (aPTT), thrombin time (TT), fibrinogen, and D-dimer, were performed daily. Additional plasma samples were stored at −80 °C for centralized enzyme-linked immunosorbent assay (ELISA) measurements of inflammatory, endothelial, and fibrinolytic biomarkers, including interleukin-6 (IL-6), syndecan-1, von Willebrand factor (vWF) antigen and activity, antithrombin, plasminogen (PLG), α2-antiplasmin, thrombin-activatable fibrinolysis inhibitor (TAFI), thrombin–antithrombin complex (TAT), plasmin–antiplasmin complex (PAP), plasminogen activator inhibitor-1 (PAI-1), and tissue plasminogen activator (tPA). Biomarkers constrained by logistics were assayed at T0, T2, and T4.

### 2.4. Outcomes

The primary outcome was VHA-derived fibrinolysis dynamics: LT and LOT (TPA- and ECA-tests) and ML and CLI-45 (EX-test), measured at T0 and T1–T4. Secondary outcomes included trajectories of plasma biomarkers of coagulation system (PLG, PAP, TAFI, PAI-1, α2-antiplasmin, and tPA; T0/T2/T4), inflammatory markers (procalcitonin, CRP, ferritin, and lactate dehydrogenase [LDH]), endothelial markers (syndecan-1 and vWF antigen/activity), classical coagulation tests, fibrinogen, antithrombin, TAT, and platelet count, and their relationships with VHA parameters.

### 2.5. Statistical Analysis

All statistical analyses were performed using R statistical software (R version 4.5.1, R Core Team, 2021) [10]. Given the exploratory nature of this study and the small sample size, the primary focus was on describing trends and patterns rather than establishing causal relationships. Descriptive statistics were used to summarize categorical variables as counts and percentages, while continuous variables were expressed as mean ± SD or median with IQR, depending on normality. No imputation, winsorization, or outlier deletion was applied and analyses used available cases.

Paired comparisons across timepoints were performed using the Wilcoxon signed-rank test, applying the Hodges–Lehmann median difference and 95% confidence intervals with false discovery rate adjustment.

For each biomarker and timepoint, box-and-jitter plots were generated to display the distribution of values with individual patient measurements superimposed. Survivors were plotted as grey circles and non-survivors as red triangles. Several biomarkers exhibited pronounced right-skewed distributions; therefore, to improve visual interpretability without modifying the underlying data, predefined upper display limits were applied for selected variables. Values exceeding these limits were displayed at the axis boundary and indicated by asterisks, explicitly denoting out-of-range observations while preserving all data points. No observations were removed or altered for statistical analyses.

For fibrinolysis-related visualizations, patients were additionally stratified by fibrinolytic resistance status using the TPA-test lysis time (LT) threshold of 312 s at admission, as proposed by Coupland et al. [11].

To illustrate temporal evolution, longitudinal “spaghetti” plots were also constructed, showing individual patient trajectories with median trend lines and interquartile-range ribbons.

Due to the study design, loss to follow-up was not applicable. Statistical significance was set at *p* < 0.05.

## 3. Results

Between January 2022 and January 2023, 15 critically ill COVID-19 patients receiving IL-6 receptor blockade were included across three Hungarian ICUs. Enrollment concluded earlier than planned as national case numbers declined; results are therefore presented as an exploratory case series.

All included patients received immunomodulation therapy and were followed for the full study period. Reasons for exclusion included cases when tocilizumab was finally not administered due to physician’s decision although the patient fit inclusion criteria or the patient presented a co-infection. The baseline characteristics of the study population can be found in Table 1.

### 3.1. Primary Outcomes

Paired Wilcoxon analyses revealed nominally significant changes in four parameters: ECA-test LOT (T0 vs. T2) and EX-test CLI-45 (T0 vs. T2, T3, and T4) (*p* < 0.05; Table S2). However, none of these remained statistically significant after false discovery rate adjustment. Consequently, interpretation focused on biological trends across timepoints rather than isolated *p*-values.

### 3.2. Inflammation and Endothelium

At admission, IL-6 (49.6 [16.2–79.2] pg/mL) and CRP (180.9 [150.8–211.0] mg/L) were markedly elevated. Following IL-6 antagonist therapy, CRP declined rapidly, with a >90% reduction within five days. Circulating IL-6 increased transiently at 48 h before declining during follow-up.

Procalcitonin showed a gradual decline after a minor early increase, while ferritin rose during the first 24 h and then stabilized (826 µg/L at 168 h), suggesting slower resolution of macrophage activation. Leukocyte counts increased follow-up, while LDH declined but remained above normal.

**Figure 1:**
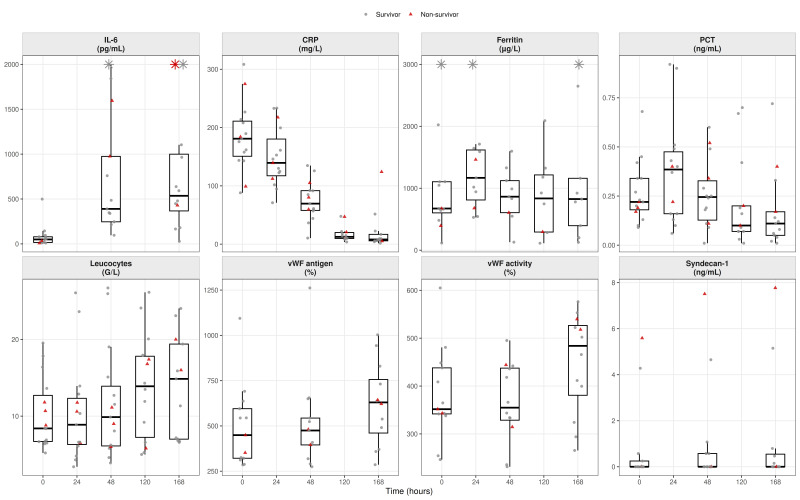
Endothelium and inflammation: upstream drivers of hemostatic shift. *Boxplots show inflammatory (IL-6, CRP, ferritin, procalcitonin, and leukocytes) and endothelial injury markers (vWF antigen/activity and syndecan-1). Boxes represent median and interquartile range; whiskers indicate 1.5 × IQR. Individual patient values are overlaid (grey circles = survivors; red triangles = non-survivors). Data are shown across timepoints (T0–T4). Values exceeding prespecified display ranges (IL-6 > 2000 pg/mL; CRP > 300 mg/L; and ferritin > 3000 µg/L) are indicated by star symbols at the upper axis boundary*.

vWF activity (351.7 [341.8–438.0] %) and antigen (449.3 [321.6–595.3] %) were markedly elevated, indicating endothelial injury, and rose further to 483.9% and 630.0% at 168 h, respectively. Syndecan-1 was generally low or undetectable, with sporadic peaks reflecting discrete glycocalyx shedding (Figure 1).

### 3.3. Coagulation Initiation and Anticoagulant Activity

Baseline viscoelastic measurements showed mildly prolonged EX-test clotting time (CT; 73 [54–88] s) and IN-test CT near the upper reference range (160 [148–182] s), indicating a modest delay in clot initiation. During follow-up, EX-test CT progressively shortened toward normal values, whereas IN-test CT remained largely unchanged, reflecting preserved extrinsic and intrinsic pathway activation despite ongoing anticoagulation.

**Figure 2:**
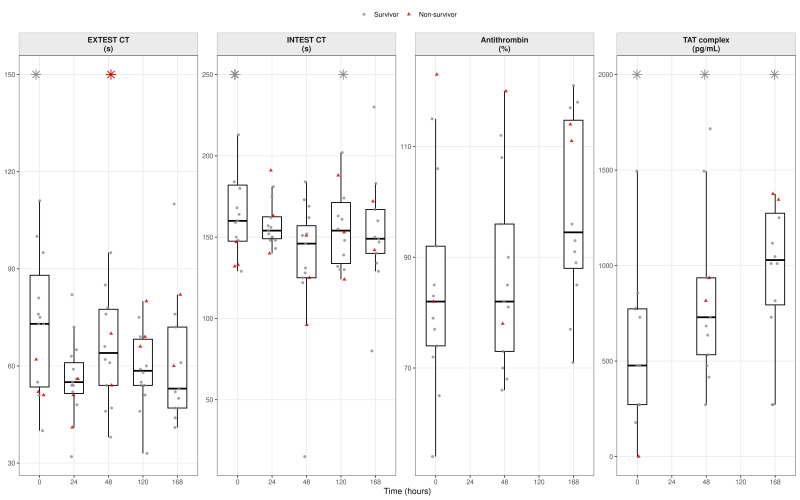
Coagulation initiation and thrombin generation. *Boxplots show viscoelastic markers of coagulation initiation (EX-test and IN-test clotting time) and biochemical markers of thrombin regulation (antithrombin activity and thrombin–antithrombin complex). Boxes represent the median and interquartile range; whiskers indicate 1.5 × IQR. Individual patient values are overlaid (grey circles = survivors; red triangles = non-survivors). Data are shown across timepoints (T0–T4). Values exceeding prespecified display ranges (EX-test CT > 150 s; IN-test CT > 250 s; and TAT complex > 2000 pg/mL) are indicated by star symbols at the upper axis boundary*.

Markers of thrombin generation were elevated at admission, with increased thrombin–antithrombin (TAT) complexes (476.8 [272.1–773.3] pg/mL) that persisted with a slight increase during follow-up. Antithrombin was mildly reduced (~80%) initially and increased over time, consistent with recovery of endogenous anticoagulant activity as inflammation resolved (Figure 2).

### 3.4. Clot Propagation and Strength

At baseline, platelet counts were within the lower-normal range (195 [180–218] G/L), while fibrinogen concentrations were markedly elevated (5.5 [5.1–6.4] g/L). Platelet counts increased modestly during follow-up (270 [220–312] G/L at 48 h) and remained stable thereafter. Fibrinogen declined steadily to 2.60 [2.12–2.96] g/L by day 7, paralleling the CRP decrease and signaling resolution of acute-phase synthesis.

**Figure 3:**
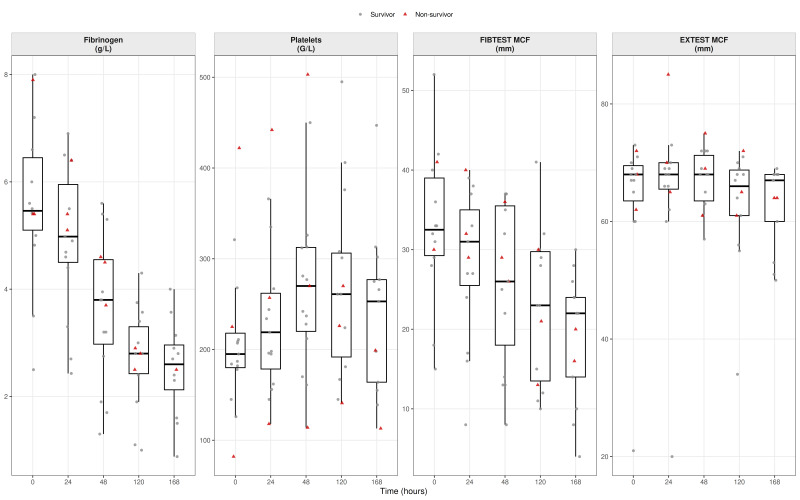
Clot propagation and strength. *Boxplots show substrates of clot formation (fibrinogen concentration and platelet count) and viscoelastic measures of clot strength (FIB-TEST and EX-test maximum clot firmness). Boxes represent the median and interquartile range; whiskers indicate 1.5 × IQR. Individual patient values are overlaid (grey circles = survivors; red triangles = non-survivors). Data are shown across timepoints (T0–T4)*.

Baseline viscoelastic measurements were consistent with this profile, showing elevated clot firmness on both EX-test and FIB-test maximum clot firmness (MCF) (EX-test: 68 [63.5–69.5] mm; FIB-test: 32.5 [29.3–39.0] mm), indicating formation of strong, fibrin-rich clots. During follow-up, EX-test MCF remained largely preserved, whereas FIB-test MCF declined to 22.0 [14.0–24.0] mm by day 7, representing an approximate 30% reduction, paralleling the decrease in fibrinogen concentration. These findings indicate a gradual re-equilibration of clot composition driven predominantly by changes in the fibrin component, while platelet-dependent clot firmness remained stable (Figure 3).

### 3.5. Fibrinolytic Activity

The fibrinolytic profile showed dynamic but non-linear evolution over the observation period. Plasminogen and α_2_-antiplasmin decreased modestly from baseline to day 7 (plasminogen: 90.0 [77.0–96.0] to 87.0 [71.5–92.0]%; α_2_-antiplasmin: 110.0 [107.0–116.0] to 100.0 [90.8–106.0]%). TAFI declined early at 48 h and partially recovered by day 7. In contrast, plasminogen activator inhibitor-1 (PAI-1) increased steadily throughout follow-up (3.42 [2.70–4.90] to 6.29 [5.40–6.83] ng/mL). Plasmin–antiplasmin (PAP) complexes increased modestly toward day 7, while D-dimer concentrations rose markedly, peaking around day 5 before slightly declining by day 7.

**Figure 4:**
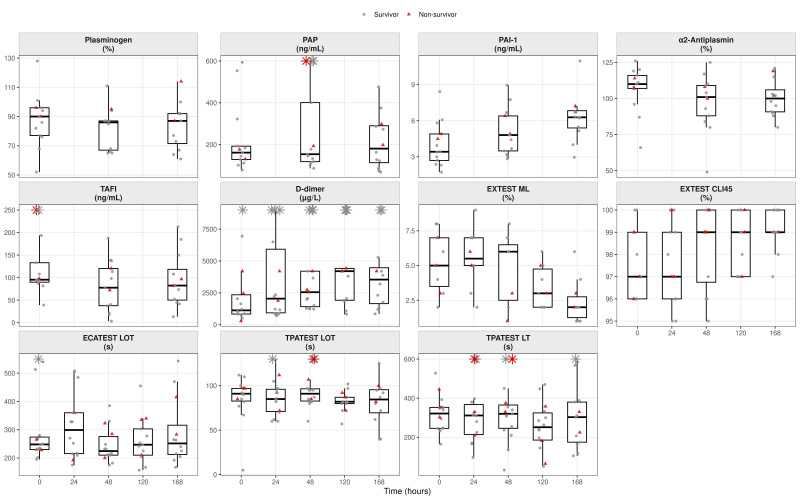
Fibrinolysis-related biomarkers and viscoelastic parameters. *Boxplots depict fibrinolytic parameters derived from viscoelastic hemostatic assays, including EX-test maximum lysis (ML), EX-test clot lysis index at 45 min (CLI45), ecarin clotting assay lysis onset time (ECA LOT), and tissue plasminogen activator (TPA)-challenge lysis onset (TPA LOT) and lysis time (TPA LT). Data are shown across timepoints (T0–T4). Values exceeding prespecified display ranges (PAP > 300 ng/mL, TAFI > 250 ng/mL, D-dimer > 7500 µg/L, ECA-test > 500 s, TPA-test LOT > 150 s, and TPA-test LT > 600 s) are indicated by star symbols at the upper axis boundary*.

These biochemical patterns were reflected in viscoelastic findings. On the EX-test, maximum lysis (ML) decreased from approximately 5% at baseline to around 2% during follow-up, while the clot lysis index at 45 min (CLI-45) remained relatively stable, indicating persistent resistance to spontaneous clot breakdown. ECA-test lysis onset time (LOT) showed a transient prolongation at 24 h before returning toward baseline values by day 7. Under tissue plasminogen activator challenge, lysis time (LT) exhibited a biphasic pattern, with early prolongation followed by shortening from approximately day 5 onward, whereas TPA-test LOT remained stable. Together, these findings indicate preserved overall fibrinolytic capacity with delayed activation (Figure 4).

Inter-individual variability and within-patient trends were illustrated using longitudinal ribbon plots with median overlays, with additional stratification by fibrinolytic resistance status at admission (Supplementary Figures S1–S11).

## 4. Discussion

This case series provides an in-depth viscoelastic and biochemical characterization of coagulation and fibrinolysis dynamics in critically ill COVID-19 patients treated with tocilizumab. Despite rapid suppression of systemic inflammation, our findings reveal a dissociation between cytokine blockade and persistent endothelial dysfunction and hemostatic imbalance, suggesting that restoration of vascular homeostasis may extend beyond IL-6 inhibition alone. These exploratory results should be interpreted with caution due to the limited sample size and hypothesis-generating design.

At baseline, patients showed inflammatory activation with endothelial-associated hemostatic imbalance; following immunomodulation, systemic inflammation subsided, whereas markers of endothelial injury continued to rise.

Physiologically, the endothelium maintains vascular homeostasis through anticoagulant, anti-inflammatory, and antioxidant pathways; however, in COVID-19 it rapidly transitions to a dysfunctional, proinflammatory, and prothrombotic phenotype. Excessive IL-6 signaling disrupts endothelial integrity, increases permeability, and promotes oxidative stress and vasoconstriction, leading to structural injury and impaired repair. Accelerated endothelial detachment and impaired regeneration may therefore contribute to sustained vascular injury and delayed recovery [12]. Recent studies have shown that endothelial dysfunction can persist well beyond the acute phase, with ongoing endothelial activation, immune-mediated injury, and sustained vascular stiffness months after recovery, underscoring the long-term vascular sequelae of COVID-19 [13,14].

Following IL-6 receptor blockade, coagulation parameters indicated partial re-equilibration of the hemostatic system. Thrombin generation markers (TAT complexes) rose modestly, while antithrombin activity increased, consistent with ongoing but controlled thrombin formation without consumption of endogenous anticoagulants. This pattern is consistent with attenuation of IL-6-driven inflammation, with endothelial injury remaining a predominant driver of thrombin generation. Consequently, antithrombin is preserved unlike in bacterial sepsis, where intense and sustained thrombin production leads to marked depletion. This profile appears characteristic of COVID-19-associated coagulopathy, with IL-6 blockade amplifying the distinction rather than causing it [15].

The kinetics of platelets and fibrinogen, together with corresponding viscoelastic patterns, indicated a reduction in the fibrin contribution to clot firmness that was offset by preserved platelet-dependent clot strength. This profile is compatible with persistent endothelial activation and platelet involvement, which may also contribute to elevated PAI-1 levels, thereby reinforcing antifibrinolytic pressure and maintaining thrombotic risk [16].

With respect to fibrinolytic balance, the observed patterns were consistent with persistent antifibrinolytic activity rather than complete fibrinolytic failure. TAFI and plasminogen declined modestly, while D-dimer concentrations increased during the first five days before slightly decreasing by day seven, indicating ongoing fibrin turnover. A modest rise in plasmin–antiplasmin (PAP) complexes further suggests limited but continuous plasmin generation.

From a viscoelastic perspective, it is important to recognize that ML and related whole-blood indices should not be interpreted as isolated markers of plasmin-mediated fibrinolysis. Tissue-factor-triggered assays contain cellular components, and post-MCF decreases in clot firmness represent a composite of enzymatic fibrinolysis and cell-mediated clot contraction. Platelet-driven contraction markedly alters clot architecture, permeability, and mechanical stability, thereby modifying the apparent “lysis” behavior recorded by viscoelastic devices. Mechanistic studies have demonstrated that clot contraction can mimic reduced fibrinolysis or, conversely, mask ongoing plasmin activity [17,18]. Consequently, low ML values or “shutdown-like” patterns in cellular assays should be interpreted with caution, as they may arise in part from altered clot-contraction dynamics rather than true suppression of fibrinolysis.

Consistent with these considerations, viscoelastic parameters showed overall stability of clot lysis indices and a non-linear evolution of TPA-test lysis time, characterized by delayed but preserved fibrinolytic capacity. IL-6 blockade was associated with attenuation of inflammation and hypercoagulability without reactivation of pathological fibrinolysis, indicating modulation rather than normalization of the hemostatic imbalance.

Interestingly, patients with fibrinolytic resistance at admission exhibited a distinct hemostatic profile during follow-up, with slightly lower vWF levels, higher fibrinogen and platelet counts, and viscoelastic features consistent with enhanced clot propagation and mechanical stability. In contrast, patients without fibrinolytic resistance showed higher D-dimer levels, suggesting more active fibrin turnover. This dichotomy aligns with the “fibrinolytic shutdown” phenotype described in severe systemic inflammation, where dense fibrin architecture and antifibrinolytic pressure coexist with low circulating fibrin degradation products [19].

Our observations are consistent with microscopic studies demonstrating that plasma clots from COVID-19 patients exhibit increased fibrin fiber density, reduced pore size, and occasionally thicker fibers, rendering clots less susceptible to enzymatic lysis. Dense fibrin networks impede diffusion of plasminogen and fibrinolytic enzymes, while elevated fibrinogen accelerates polymerization and fiber packing. In parallel, endothelial injury, neutrophil extracellular traps, and high thrombin flux promote extensive cross-linking and tighter fibrin alignment. Together, these processes generate compact, lysis-resistant clots that persist within a pro-inflammatory and pro-coagulant milieu, amplifying thrombotic risk in COVID-19 [20,21,22,23].

Several clinical studies corroborate our findings. Di Nisio et al. reported rapid improvements in coagulation parameters following a single subcutaneous dose of tocilizumab in hospitalized COVID-19 patients, with reductions in D-dimer and fibrinogen, rising platelet counts, and shortened PT/aPTT concurrent with improved respiratory function [24]. Conversely, Chan et al. observed transient D-dimer surges after tocilizumab, particularly in patients not receiving full-dose anticoagulation, suggesting transient unmasking of latent fibrinolytic activity and release of clot-bound degradation products [25]. More recently, Aljuhani et al. demonstrated in a large propensity-matched ICU cohort that early tocilizumab use was associated with lower fibrinogen trajectories and improved survival without excess thrombosis, suggesting an overall hemostatic benefit [26]. Similarly, Nagy et al. found divergent fibrinolytic responses, with tocilizumab-treated patients showing shorter tPA-ROTEST lysis times compared with controls and increasing PAI-1 levels over time, whereas control and dexamethasone groups showed the opposite pattern [27]. The randomized Coag-ImmCovA trial further showed that, by day 29, tocilizumab reduced hypercoagulability (prolonged CFT and reduced MCF) and decreased fibrinogen compared with anakinra or standard care. Structural analysis revealed denser fibrin networks early after treatment, followed by remodeling with larger pores and thicker fibers, indicating gradual reorganization of the clot matrix [28].

### Strengths and Limitations

The novelty of this study lies in the detailed temporal characterization of inflammatory, coagulation, endothelial, and fibrinolytic dynamics following IL-6 receptor blockade.

Nevertheless, the findings presented here must be interpreted cautiously, given several inherent limitations. Primarily, this investigation was an observational pilot study involving a small number of critically ill COVID-19 patients at a late pandemic stage, when widespread vaccination and prior viral exposures may have modified patient phenotypes and immune responses. Thus, extrapolation of our results to earlier pandemic waves or unvaccinated populations warrants caution. Patient selection was pragmatic and may introduce selection bias. Additionally, the absence of a control group and reliance on before-and-after comparisons further limit the robustness and causal interpretability of our results. Consequently, our findings should be viewed as hypothesis-generating rather than definitive evidence. Finally, concomitant therapies, including corticosteroids and anticoagulation, represent unavoidable confounders inherent to standard-of-care management in severe COVID-19.

## 5. Conclusions

Taken together, our findings describe parallel temporal patterns in inflammatory, endothelial, and coagulation parameters following IL-6 antagonism. While inflammatory markers declined, indices of endothelial activation and hemostatic imbalance persisted in several patients. These exploratory observations highlight the complexity of COVID-19-associated coagulopathy and support further investigation into mechanisms beyond cytokine-mediated inflammation.

## Figures and Tables

**Table 1:** General characteristics of the study population at admission.

Characteristic	Value
Study population	15
Male	12/15 (80%)
Age	68.3 (8.6); 69.0 [64.0, 74.0]
BMI	32.2 (6.2); 30.0 [27.2, 35.4]
Hypertension	15/15 (100.0%)
Ischemic heart disease	6/15 (40.0%)
Atrial fibrillation	4/15 (26.6%)
Diabetes Type II	7/14 (50.0%)
Chronic Respiratory Disease	2/15 (13.3%)
Autoimmune disease	2/15 (13.3%)
Malignant Disease	1/15 (6.7%)
Renal disease	1/15 (6.7%)
Neurological or psychiatric disease	3/15 (20.0%)
Obesity	7/15 (46.7%)
APACHE II	11.2 (3.4); 12.0 [8.2, 13.0]
Clinical frailty scale	3.5 (1.2); 3.0 [3.0, 4.0]
Severe organ failure at admission	7/15 (46.7%)
Vasopressor support	4/15 (26.7%)
Currently on oxygen or ventilated	15/15 (100.0%)
Type of respiratory support	
High-Flow Nasal Cannula	4/15 (26.7%)
Invasive Mechanical Ventilation	6/15 (40.0%)
Non-Invasive Ventilation	3/15 (20.0%)
Non-rebreather mask	2/15 (13.3%)
Prone position	7/15 (46.7%)
Neuromuscular relaxant	2/15 (13.3%)
PaO_2_	74.4 (21.2); 69.2 [60.4, 80.2]
FiO_2_	0.9 (0.2); 1.0 [0.8, 1.0]
PaO_2_/FiO_2_ (Horowitz index)	91 (40); 74 [65, 114]
PaCO_2_ (mmHg)	39.4 (6.4); 36.6 [35.1, 43.9]
Ferritin (ug/L)	1351.2 (1930.1); 673.0 [602.8, 1105.2]
CRP (mg/L)	183.7 (58.6); 180.9 [150.8, 211.0]
LDH (U/L)	841.4 (396.1); 759.5 [531.2, 1176.0]
Leucocytes (G/L)	10 (5); 8 [7, 13]
Hemoglobin (g/L)	127 (20); [120, 111, 146]
Hematocrit (%)	37 (5); 36 [33, 42]
Thrombocytes (G/L)	209 (81); 195 [180, 218]
ASAT/GOT (U/L)	76.3 (45.6); 62.0 [42.0, 109.5]
ALAT/GPT (U/L)	47.7 (33.1); 31.0 [26.5, 64.5]
APTT (s)	39.0 (6.5); 40.4 [36.0, 44.7]
Thrombin time (s)	18.8 (2.9); 18.1 [17.4, 21.6]
Fibrinogen (g/L)	5.6 (1.5); 5.5 [5.1, 6.4]
D dimer (ug/L)	10,674.9 (33,130.4); 1125.5 [837.5, 2347.5]
Tocilizumab dose (mg)	704.0 (98.9); 720.0 [600.0, 800.0]
Corticosteroids	15/15 (100.0%)
Antiviral medication (acyclovir, remdesivir, or both)	14/15 (93.3%)
Blood products or derivatives	0/15 (0.0%)
Immunoglobulin	0/15 (0.0%)
Antiplatelet agents	8/15 (53.3%)
Aspirin	8/15 (53.3%)
Clopidogrel	2/15 (13%)
Anticoagulants	13/15 (86.7%)
DOAC	1/13 (7.7%)
LMWH	12/13 (92.3%)
Daily dose of LMWH (IU)	11,500.0 (5125.7); 12,000.0 [8000.0, 13,000.0]
Admission source to ICU	Emergency department: 12 (80%); Hospital ward: 3 (20%)
Patient outcome at the end of the study period (7 days)	
Deceased	3/15 (20.0%)
Remained in ICU	8/15 (53.3%)
Discharged from ICU and transferred to ward	4/15 (26.7%)

Continuous: Mean (SD); Median [IQR]; Binary: n/N, (%).

## Data Availability

The raw data supporting the conclusions of this article will be made available by the authors on request.

## Supplementary Materials

The following supporting information can be downloaded at: https://www.mdpi.com/article/10.3390/biomedicines14010254/s1; Figure S1: Temporal evolution of endothelial and inflammatory biomarkers (boxplots); Figure S2: Temporal evolution of coagulation initiation and thrombin generation parameters (boxplots); Figure S3: Temporal evolution of clot propagation and mechanical clot strength parameters (boxplots); Figure S4: Temporal evolution of fibrinolytic and thrombin-regulatory biomarkers (boxplots); Figure S5: Temporal evolution of viscoelastic fibrinolysis parameters (boxplots); Figure S6: Longitudinal ribbon plots (log_10_ scale) of endothelial and inflammatory biomarkers; Figure S7: Longitudinal ribbon plots (log_10_ scale) of coagulation initiation and thrombin generation parameters; Figure S8: Longitudinal ribbon plots (log_10_ scale) of clot propagation and mechanical strength parameters; Figure S9: Longitudinal ribbon plots (log_10_ scale) of fibrinolytic and thrombin-regulatory biomarkers (excluding tPA); Figure S10: Longitudinal ribbon plots (log_10_ scale) of viscoelastic fibrinolysis indices; Figure S11: Complete viscoelastic hemostatic assay panel, including FIBTEST, stratified by survival status; Table S1: Detailed general characteristics of the study population; Table S2: Paired Wilcoxon signed-rank analyses with Hodges–Lehmann estimators and Benjamini–Hochberg false discovery rate correction for primary and secondary viscoelastic fibrinolysis outcomes; Table S3: Trajectories of inflammation and hemostasis parameters across time points (T0–T4); Table S4: ClotPro viscoelastic assays and parameters: physiological basis and interpretive framework; Table S5: Normal values on ClotPro as indexed by manufacturer; Table S6: STROBE Statement–checklist of items that should be included in reports of observational studies.

## Abbreviations

The following abbreviations are used in this manuscript:

aPTT	Activated Partial Thromboplastin Time
CFT	Clot Formation Time
CLI-30/45	Clot Lysis Index at 30 or 45 min
COVID-19	Coronavirus Disease 2019
CRP	C-reactive protein
CT	Clotting Time
D-dimer	Fibrin degradation product
ECA-test	Ecarin Clotting Assay
eCRF	Electronic Case Report Form
ELISA	Enzyme-Linked Immunosorbent Assay
EX-test	Extrinsic Pathway Test
FDR	False Discovery Rate
FIBTEST	Fibrinogen Test
FiO_2_	Fraction of Inspired Oxygen
FR	Fibrinolytic Resistance
G/L	Giga per Liter
HFNO	High-Flow Nasal Oxygen
ICU	Intensive Care Unit
IL-6	Interleukin-6
INR	International Normalized Ratio
IN-test	Intrinsic Pathway Test
LOT	Lysis Onset Time
LT	Lysis Time
MCF	Maximum Clot Firmness
ML	Maximum Lysis
NIV	Non-invasive ventilation
PaCO_2_	Partial Pressure of Carbon Dioxide
PaO_2_	Partial Pressure of Oxygen in Arterial Blood
PAI-1	Plasminogen Activator Inhibitor-1
PAP	Plasmin–Antiplasmin Complex
PCT	Procalcitonin
PCR	Polymerase Chain Reaction
PLG	Plasminogen
RVVTEST	Russell Viper Venom Test
sIL-6R	Soluble Interleukin-6 Receptor
SIRS	Systemic Inflammatory Response Syndrome
TAT	Thrombin–Antithrombin Complex
TAFI	Thrombin-Activatable Fibrinolysis Inhibitor
Tpa	Tissue Plasminogen Activator
TPA-test	tPA-Enhanced Lysis Test
TT	Thrombin Time
VHA	Viscoelastic Hemostatic Assay
vWF	von Willebrand Factor
vWF:Act	von Willebrand Factor Activity
vWF:Ag	von Willebrand Factor Antigen
