# Supplementary material for: Exploratory Analysis of Coagulation and Fibrinolysis Trajectories After IL-6 Antagonist Therapy in COVID-19: A Case Series
Source: Biomedicines. 2026 Jan 22;14(1):254. doi: 10.3390/biomedicines14010254 (PMC12838625; doi:10.3390/biomedicines14010254)
Supplement: Supplementary file 1 [file biomedicines-14-00254-s001.zip › biomedicines-4049317-supplementary.pdf]

Supplementary Materials

Table of contents

I. Supplementary Methods and Descriptive Data

1. Supplementary Text S1. Study Population
2. Table S1. Detailed Baseline Characteristics of the Study Population
3. Table S2. Paired Wilcoxon Analysis with Hodges–Lehmann Estimates and FDR Correction for Primary Outcomes
4. Table S3. Trajectories of Inflammatory, Endothelial, Coagulation, and Fibrinolysis Parameters across Timepoints (T0–T4)

II. Supplementary Figures – Boxplots

1. Figure S1. Temporal trajectories of inflammatory and endothelial injury markers (boxplots)
2. Figure S2. Temporal evolution of coagulation initiation and thrombin generation markers (boxplots)
3. Figure S3. Temporal evolution of clot formation and mechanical strength parameters (boxplots)
4. Figure S4. Temporal evolution of fibrinolysis-related and thrombin-regulatory biomarkers (boxplots)
5. Figure S5. Temporal evolution of viscoelastic fibrinolysis parameters (boxplots)

III. Supplementary Figures – Spaghetti/Ribbon plots

1. Figure S6. Longitudinal trajectories of inflammatory and endothelial injury markers
2. Figure S7. Longitudinal trajectories of coagulation initiation and thrombin generation parameters
3. Figure S8. Longitudinal trajectories of clot formation and mechanical strength parameters
4. Figure S9. Longitudinal trajectories of fibrinolysis-related biomarkers
5. Figure S10. Longitudinal trajectories of viscoelastic fibrinolysis parameters

IV. Supplementary References and Methodological Tables

1. Table S4. ClotPro® Viscoelastic Assays and Parameters: Physiological Basis and Interpretive Framework
2. Table S5. Manufacturer-Reported Reference Ranges for ClotPro® Parameters
3. Supplementary Methods S2. Laboratory Assays and ELISA Characteristics
4. Table S6. STROBE Checklist for Observational Studies

Supplementary Text S1 Study population

The study population consisted of 15 patients, mostly male, with a mean age of 68.3 ± 8.6 years. All patient presented with hypertension and four with atrial fibrillation, while diabetes was reported in 7 cases. Chronic respiratory disease (emphysema and COPD Gold I) and autoimmune conditions (Hashimoto thyroiditis) were observed in 13.3% each, and respectively one patient had chronic kidney disease (Gr. II) and another a history of malignancy (prostate cancer). The mean APACHE II score was 11.2 ± 3.4. Standard care included corticosteroids and anticoagulation; most also received antivirals such as acyclovir and remdesivir.

At baseline, patients demonstrated markedly elevated inflammatory parameters. Viscoelastic testing at T0 showed delayed clot initiation (prolonged EX- and RVV-CT) probably from already being on anticoagulation with elevated fibrinogen contribution (FIB-MCF above reference) and high-normal overall clot firmness (MCF). Routine lysis indices (ML, CLI-30/45) were largely within reference, whereas the TPA-test lysis time was prolonged (median of 322s, with half of patients exceeding the ≥300 s threshold which is consistent with decreased fibrinolytic response according to Coupland et al. [1].

All patients required oxygen therapy with a median PaO_2/FiO_2 ratio of 73.1 [IQR 0.469, 192], reflecting severe respiratory failure and were administered corticosteroids, anticoagulation and antivirals. Through the 7 days, 1 out of 15 patient died; by day 30, cumulative mortality was 3out of 15. Five patients were discharged from the ICU during follow-up. Baseline characteristics are summarized in Table 1.

Table S1. Detailed general characteristics of the study population

Characteristic	Overall
N	15
Statistics key	Mean (SD) ; Median [IQR] ; Missing: n/N (%)
**Study centers**	
Institute	
Szent György Univ. Hosp., Anesth. & ICU Dept. (Székesfehérvár)	6/15 (40.0%)
Univ. of Pécs, Clin. Centre, Anesth. & ICU Dept. (Pécs)	3/15 (20.0%)
Flór Ferenc Hosp. Anesth. & ICU Dept. (Kistarcsa)	6/15 (40.0%)
**Demographics and Comorbidities**	
Female	3/15 (20.0%)
Male	12/15 (80.0%)
Age	68.3 (8.6) ; 69.0 [64.0, 74.0] ;
BMI	32.2 (6.2) ; 30.0 [27.2, 35.4] ;
Hypertension	15/15 (100.0%)
Ischemic heart disease	6/15 (40.0%)
Atrial fibrillation	4/15 (26.6%)
Diabetes Type II	7/14 (50.0%)
Chronic Respiratory Disease	2/15 (13.3%)
Autoimmune disease	2/15 (13.3%)

Malignant Disease<br>Renal disease<br>Neurological or psychiatric disease	1/15 (6.7%)<br>1/15 (6.7%)<br>3/15 (20.0%)
Obesity	7/15 (46.7%)
**Severity at admission**	
APACHE II	11.2 (3.4) ; 12.0 [8.2, 13.0] ; Missing: 1/15 (6.7%)
Clinical frailty scale	3.5 (1.2) ; 3.0 [3.0, 4.0]
Severe organ failure at admission	7/15 (46.7%)
Vasopressor support	4/15 (26.7%)
**Respiratory support**	
Currently on oxygen or ventilated	15/15 (100.0%)
Type of respiratory support	
High Flow Nasal Cannula	4/15 (26.7%)
Invasive Mechanical Ventilation	6/15 (40.0%)
Non Invasive Ventilation	3/15 (20.0%)
Non-rebreather mask	2/15 (13.3%)
Prone position	7/15 (46.7%)
Neuromuscular relaxant	2/15 (13.3%)
PaO2	74.4 (21.2) ; 69.2 [60.4, 80.2] ; Missing: 1/15 (6.7%)
FiO2	0.9 (0.2) ; 1.0 [0.8, 1.0] ; Missing: 1/15 (6.7%)
PaO2/FiO2 (Horowitz index)	91 (40) ; 74 [65, 114] ; Missing: 1/15 (6.7%)
PaCO2 (mmHg)	39.4 (6.4) ; 36.6 [35.1, 43.9]
**Laboratory values**	
Ferritin (ug/L)	1351.2 (1930.1) ; 673.0 [602.8, 1105.2] ; Missing: 3/15 (20.0%)
CRP (mg/l)	183.7 (58.6) ; 180.9 [150.8, 211.0]
LDH (U/l)	841.4 (396.1) ; 759.5 [531.2, 1176.0] ; Missing: 1/15 (6.7%)
Leucocytes (G/l)	10 (5) ; 8 [7, 13]
Hemoglobin (g/l)	127 (20) ; 120 [111, 146]
Hematocrite (%)	37 (5) ; 36 [33, 42] ;
Thrombocytes (G/l)	209 (81) ; 195 [180, 218] ;
ASAT / GOT (U/l)	76.3 (45.6) ; 62.0 [42.0, 109.5] ;
ALAT / GPT (U/l)	47.7 (33.1) ; 31.0 [26.5, 64.5] ;
APTT (sec)	39.0 (6.5) ; 40.4 [36.0, 44.7] ; Missing: 2/15 (13.3%)
Thrombin time (sec)	18.8 (2.9) ; 18.1 [17.4, 21.6] ; Missing: 2/15 (13.3%)
Fibrinogen (g/l)	5.6 (1.5) ; 5.5 [5.1, 6.4] ; Missing: 1/15 (6.7%)
D dimer (ug/L)	10674.9 (33130.4) ; 1125.5 [837.5, 2347.5] ; Missing: 1/15 (6.7%)
INR	1.2 (0.2) ; 1.1 [1.0, 1.2] ;
**Baseline ClotPro parameters**	
EXTEST CT (s)	84 (53) ; 73 [54, 88] ;
EXTEST CFT (s)	145 (359) ; 50 [46, 56] ;
EXTEST alpha angle (deg)	75.8 (9.6) ; 79.0 [77.5, 79.5] ;
EXTEST MCF (mm)	64.1 (12.6) ; 68.0 [63.5, 69.5] ;
EXTEST ML (%)	5.3 (2.1) ; 5.0 [3.5, 7.0] ; Missing: 4/15 (26.7%)
EXTEST LOT (s)	208 (62) ; 192 [176, 212] ;
EXTEST CLI 30 (%)	99.5 (0.6) ; 100.0 [99.0, 100.0] ;
EXTEST CLI 45 (%)	97.6 (1.6) ; 97.0 [96.0, 99.0] ; Missing: 3/15 (20.0%)
INTEST CT (s)	204 (123) ; 160 [148, 182] ;
INTEST CFT (s)	64 (22) ; 58 [53, 60] ; Missing: 1/15 (6.7%)
INTEST alpha angle (deg)	73.2 (16.0) ; 78.0 [77.5, 79.5] ;
INTEST MCF (mm)	61.5 (13.0) ; 64.0 [61.0, 67.0] ;
INTEST ML (%)	11.8 (24.1) ; 5.0 [3.0, 8.0] ; Missing: 3/15 (20.0%)
FIBTEST CT (s)	123 (99) ; 100 [79, 123] ; Missing: 1/15 (6.7%)
FIBTEST CFT (s)	97 (56) ; 68 [65, 104] ; Missing: 9/15 (60.0%)
FIBTEST MCF (mm)	32.9 (9.5) ; 32.5 [29.2, 39.0] ; Missing: 1/15 (6.7%)
RVVTEST CT (s)	208 (255) ; 129 [92, 158] ;
RVVTEST CFT (s)	104 (102) ; 50 [46, 114] ;
RVVTEST alpha angle (deg)	72.5 (13.2) ; 80.0 [70.0, 80.5] ;

RVVTEST MCF (mm)	60.7 (9.3) ; 64.0 [55.5, 67.5] ;
RVVTEST ML (%)	5.2 (2.3) ; 6.0 [3.2, 6.0] ; Missing: 5/15 (33.3%)
ECATEST CT (s)	76 (20) ; 70 [64, 80] ;
ECATEST CFT (s)	117 (52) ; 100 [91, 106] ;
ECATEST alpha angle (deg)	70.5 (9.1) ; 74.0 [72.5, 75.0] ;
ECATEST MCF (mm)	65.5 (8.8) ; 70.0 [64.0, 71.0] ;
ECATEST LOT (s)	309 (152) ; 248 [230, 274] ;
ECATEST CLI 30 (%)	100.0 (0.0) ; 100.0 [100.0, 100.0] ;
ECATEST CLI 45 (%)	100.0 (0.0) ; 100.0 [100.0, 100.0] ; Missing: 3/15 (20.0%)
TPATEST LT (s)	321 (95) ; 322 [246, 353] ; Missing: 1/15 (6.7%)
TPATEST ML (%)	96.1 (2.2) ; 97.0 [96.0, 97.0] ; Missing: 1/15 (6.7%)
TPATEST LOT (s)	85 (26) ; 91 [83, 97] ; Missing: 1/15 (6.7%)
TPATEST CLI 30 (%)	10.4 (19.7) ; 5.0 [4.0, 5.0] ;
TPATEST CLI 45 (%)	11.2 (22.0) ; 5.0 [4.0, 5.0] ; Missing: 3/15 (20.0%)
**Therapies & outcomes**	
Tocilizumab dose	704.0 (98.9) ; 720.0 [600.0, 800.0]
Corticosteroids	15/15 (100.0%)
Antiviral medication (acyclovir, remdesivir or both)	14/15 (93.3%)
Blood products or derivatives	0/15 (0.0%)
Immunglobulin	0/15 (0.0%)
Antiplatelet agents	8/15 (53.3%)
Aspirin	8/15 (53.3%)
Clopidogrel	Yes 2/8 (13%)
Antikoagulants	Yes 13/15 (86.7%)
DOAC	Yes 1/13 (7.7%) Missing 2/15 (13.3%)
LMWH	Yes 12/13 (92.3%) Missing 2/15 (13.3%)
Daily dose of LMWH UI	11500.0 (5125.7) ; 12000.0 [8000.0, 13000.0] ; Missing: 3/15 (20.0%)
Patient outcome	
Deceased	3/15 (20.0%)
Remained in ICU	8/15 (53.3%)
Transferred to another ward	4/15 (26.7%)
**Endothelial & coagulation biomarkers**	
Interleukin-6 (pg/mL)	83.7 (131.2) ; 49.6 [16.2, 79.2] ; Missing: 2/15 (13.3%)
Alpha-2-antiplasmin (%)	106.7 (15.9) ; 110.0 [107.0, 116.0] ; Missing: 2/15 (13.3%)
Plasminogen (%)	87.4 (18.1) ; 90.0 [77.0, 96.0] ; Missing: 2/15 (13.3%)
Antithrombin III (%)	85.2 (19.6) ; 82.0 [74.0, 92.0] ; Missing: 2/15 (13.3%)
vWF akt (%)	381.8 (95.8) ; 351.7 [341.8, 438.0] ; Missing: 2/15 (13.3%)
vWF ag (%)	495.3 (229.9) ; 449.3 [321.6, 595.3] ; Missing: 2/15 (13.3%)
TAFI (ng/mL)	171.6 (175.0) ; 95.4 [89.9, 133.1] ; Missing: 2/15 (13.3%)
TAC (pg/mL)	702.1 (623.1) ; 476.8 [272.1, 773.3] ; Missing: 2/15 (13.3%)
PAP (ng/mL)	221.7 (167.3) ; 161.8 [128.9, 192.7] ; Missing: 2/15 (13.3%)
Syndecan (ng/mL)	0.8 (1.9) ; 0.0 [0.0, 0.2] ; Missing: 2/15 (13.3%)
PAI (ng/mL)	4.0 (1.9) ; 3.4 [2.7, 4.9] ; Missing: 2/15 (13.3%)
tPA (ng/mL)	0.0 (0.0) ; 0.0 [0.0, 0.0] ; Missing: 2/15 (13.3%)
Continuous: Mean (SD) ; Median [IQR] ; Missing: n/N (%). Binary: n/N, % + Missing.	

Table S2. Paired Wilcoxon Analysis With Hodges–Lehmann Estimates and FDR Correction for primary outcomes

Variable	Comparison	n_pairs	Median_T0	Median_Tn	Delta_med	HL_shift	HL_CI	p_value	p_adj	Sig	FDR_BH
TPA-test LT (s)	T0 vs T1	13	340.0 [245.0–353.0]	313.0 [225.0–380.0]	-20	17.5	[-141.5, 84.0]	0.53	0.85		
TPA-test LT (s)	T0 vs T2	14	321.5 [245.8–353.0]	325.0 [268.2–370.0]	-25.5	6.5	[-594.5, 76.5]	0.9	0.94		
TPA-test LT (s)	T0 vs T3	13	340.0 [248.0–353.0]	263.0 [182.0–333.0]	-98	96.5	[-21.5, 151.5]	0.11	0.31		
TPA-test LT (s)	T0 vs T4	12	321.5 [247.2–353.0]	306.5 [196.5–382.0]	-44	40.4	[-91.5, 95.5]	0.48	0.85		
TPA-test LOT (s)	T0 vs T1	14	91.0 [82.8–97.0]	88.5 [74.5–96.5]	0	-0.5	[-36.0, 16.0]	0.94	0.94		
TPA-test LOT (s)	T0 vs T2	13	92.0 [85.0–97.0]	95.0 [85.0–97.0]	0	-2.5	[-25.0, 10.0]	0.69	0.86		
TPA-test LOT (s)	T0 vs T3	12	94.5 [84.2–97.8]	82.0 [80.0–88.2]	-11.5	7.5	[-5.0, 20.0]	0.21	0.52		
TPA-test LOT (s)	T0 vs T4	11	92.0 [83.5–98.5]	87.0 [76.0–96.0]	-8	8.7	[-8.0, 23.0]	0.29	0.63		
ECA-test LOT (s)	T0 vs T1	14	249.0 [230.5–277.0]	299.0 [215.5–360.0]	-20	8	[-58.0, 66.0]	0.78	0.86		
ECA-test LOT (s)	T0 vs T2	14	241.5 [230.0–267.2]	224.5 [210.0–275.8]	-30	30.5	[4.0, 109.5]	0.04	0.23	*	
ECA-test LOT (s)	T0 vs T3	13	235.0 [230.0–265.0]	247.0 [210.0–303.0]	-20	13.5	[-31.5, 53.0]	0.55	0.85		
ECA-test LOT (s)	T0 vs T4	12	241.5 [229.5–265.8]	251.5 [212.5–316.0]	-8.5	6.5	[-40.5, 43.0]	0.67	0.86		
EX-test ML (%)	T0 vs T1	10	5.5 [3.2–7.0]	5.5 [5.0–7.0]	0	-0.5	[-3.5, 2.0]	0.73	0.86		
EX-test ML (%)	T0 vs T2	7	6.0 [3.5–7.0]	6.0 [2.5–6.5]	-2	1.5	[-3.0, 3.0]	0.39	0.79		
EX-test ML (%)	T0 vs T3	8	5.5 [3.8–7.2]	3.0 [2.0–5.0]	-2.5	2.2	[-0.0, 5.0]	0.11	0.31		
EX-test ML (%)	T0 vs T4	8	5.5 [3.0–7.2]	2.0 [1.8–3.2]	-3	2.7	[-0.5, 5.0]	0.06	0.23		
EX-test CLI-45 (%)	T0 vs T1	12	97.0 [96.0–99.0]	97.5 [96.8–99.2]	0	-0.5	[-2.0, 2.0]	0.67	0.86		
EX-test CLI-45 (%)	T0 vs T2	11	97.0 [96.0–99.0]	99.0 [98.0–100.0]	1	-2	[-3.5, 0.0]	0.05	0.23	*	
EX-test CLI-45 (%)	T0 vs T3	11	97.0 [96.5–99.0]	99.0 [98.5–100.0]	1	-2	[-2.5, -1.5]	0.02	0.23	*	
EX-test CLI-45 (%)	T0 vs T4	10	97.5 [96.2–99.0]	99.0 [99.0–100.0]	1.5	-2	[-3.0, -1.0]	0.03	0.23	*	

Variable, biomarker or viscoelastic parameter analyzed; Comparison, timepoint contrast (Tn vs T0); n_pairs, number of paired observations; Median_T0, median at baseline; Median_Tn, median at follow-up; Delta_med, median change (Median_Tn – Median_T0); HL_shift, Hodges–Lehmann shift estimator of the median paired difference; HL_CI, 95% confidence interval of the Hodges–Lehmann estimator; p_value, two-sided p-value from the Wilcoxon signed-rank test; p_adj, p-value after False Discovery Rate correction (Benjamini–Hochberg); Sig, significance coding (= p<0.05, ** = p<0.01, *** = p<0.001*); FDR, False Discovery Rate adjustment procedure.*

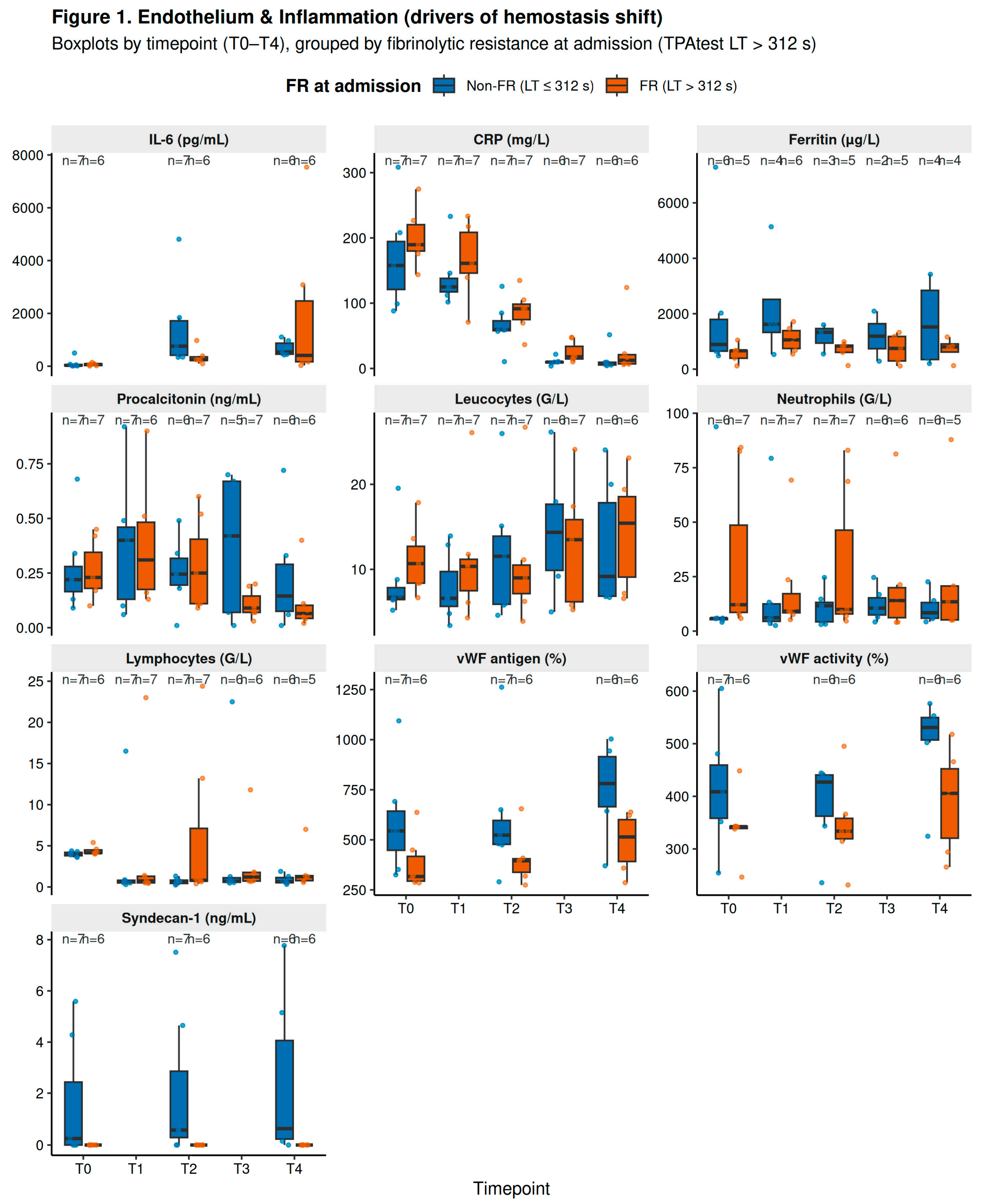

Figure S1: Boxplots display the temporal trajectories of key inflammatory (IL-6, CRP, Ferritin, Procalcitonin, Leukocytes, Neutrophils, Lymphocytes) and endothelial injury markers (vWF antigen, vWF activity, Syndecan-1) across all timepoints (T0–T4). Patients were stratified according to fibrinolytic resistance (FR) at admission, defined by a TPA-test lysis time (LT) > 312 seconds. Non-FR patients (TPA-LT ≤ 312 s) are shown in blue, and FR patients in orange. For each panel, the number of available measurements per group and timepoint is indicated (n = x). Boxplots represent medians and interquartile ranges; individual points denote raw patient-level values.

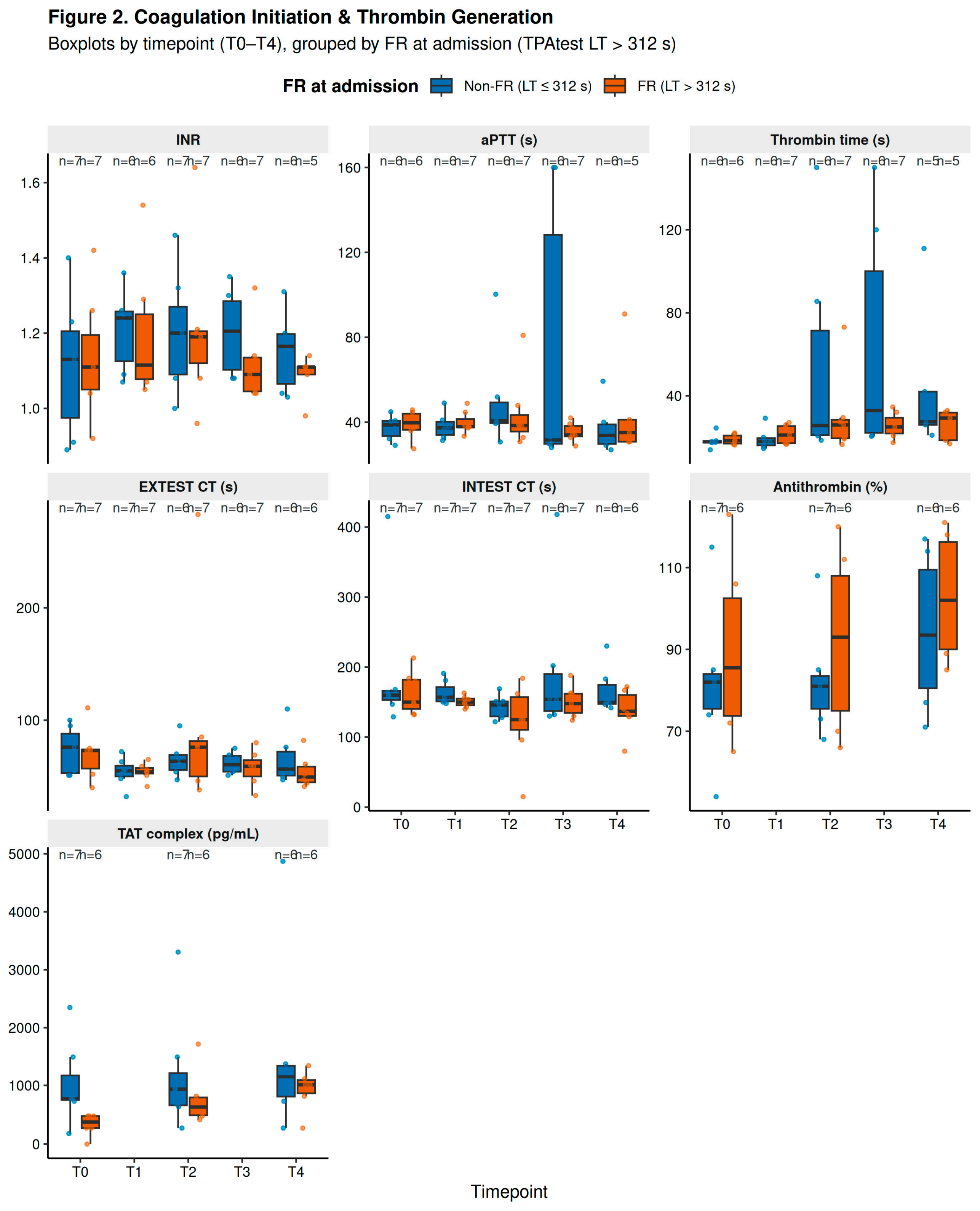

Figure S2: Boxplots illustrate the temporal evolution of coagulation initiation parameters (INR, aPTT, thrombin time), viscoelastic clotting times (EX-test CT, IN-test CT), and markers of thrombin generation (TAT complex, Antithrombin III) from T0 to T4. Patients were stratified by fibrinolytic resistance (FR) at admission, defined as TPA-test lysis time (LT) > 312 s. Non-FR patients (blue; LT ≤ 312 s) and FR patients (orange; LT > 312 s) are shown separately. Sample sizes for each timepoint and group are indicated above each panel (n = x). Boxplots represent medians and interquartile ranges, with individual data points overlaid.

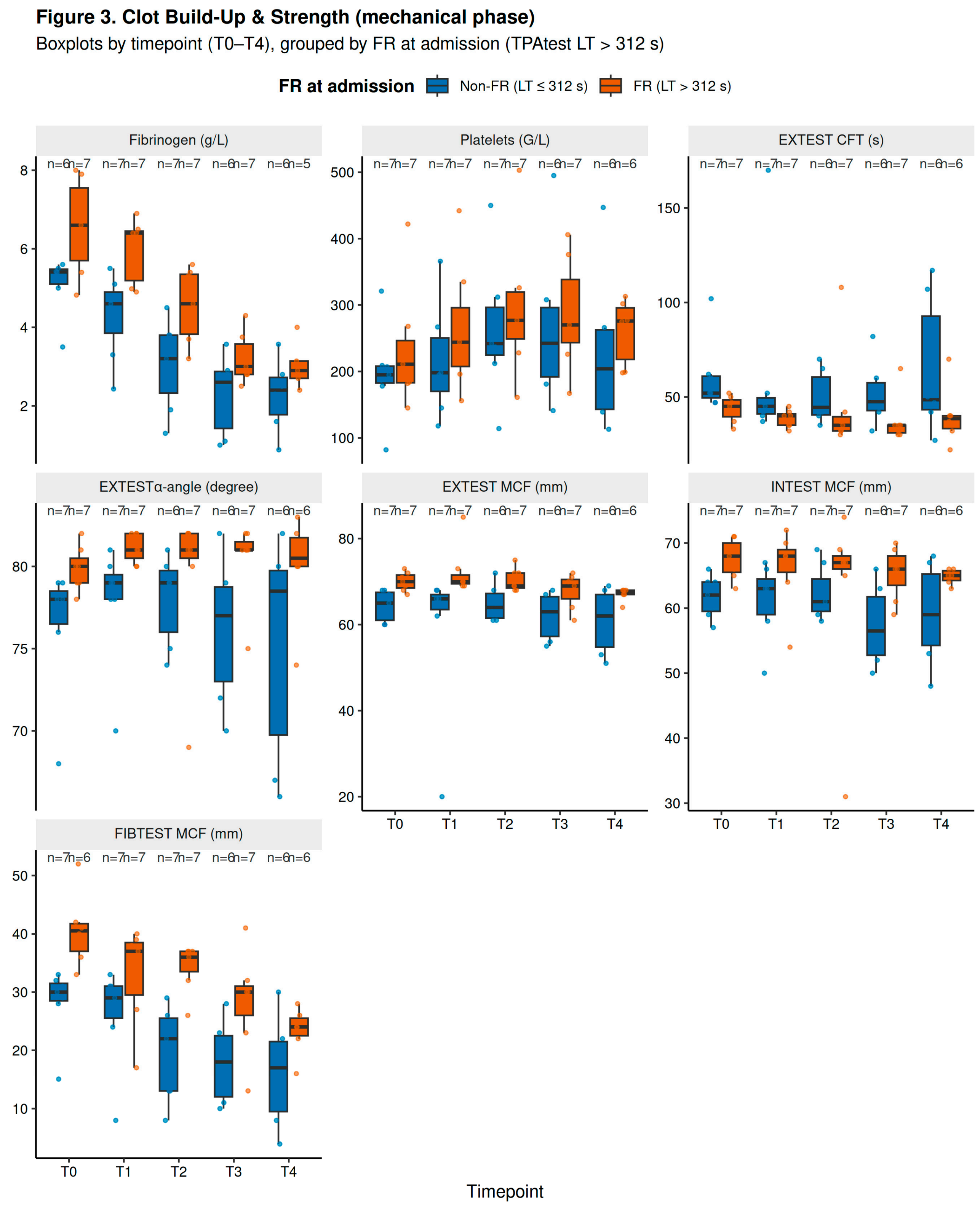

Figure S3: Boxplots illustrate the temporal evolution of parameters describing clot build-up and mechanical clot strength, including fibrinogen concentration, platelet count, EX-test CFT, EX-test α-angle, EX-test MCF, IN-test MCF, and FIB-test MCF across timepoints T0–T4. Patients were stratified by fibrinolytic resistance (FR) at admission, defined as a TPA-test lysis time > 312 seconds. Non-FR patients (blue; LT ≤ 312 s) and FR patients (orange; LT > 312 s) are shown separately, with the number of available measurements at each timepoint displayed above each panel. Boxplots represent medians and interquartile ranges with individual observations overlaid.

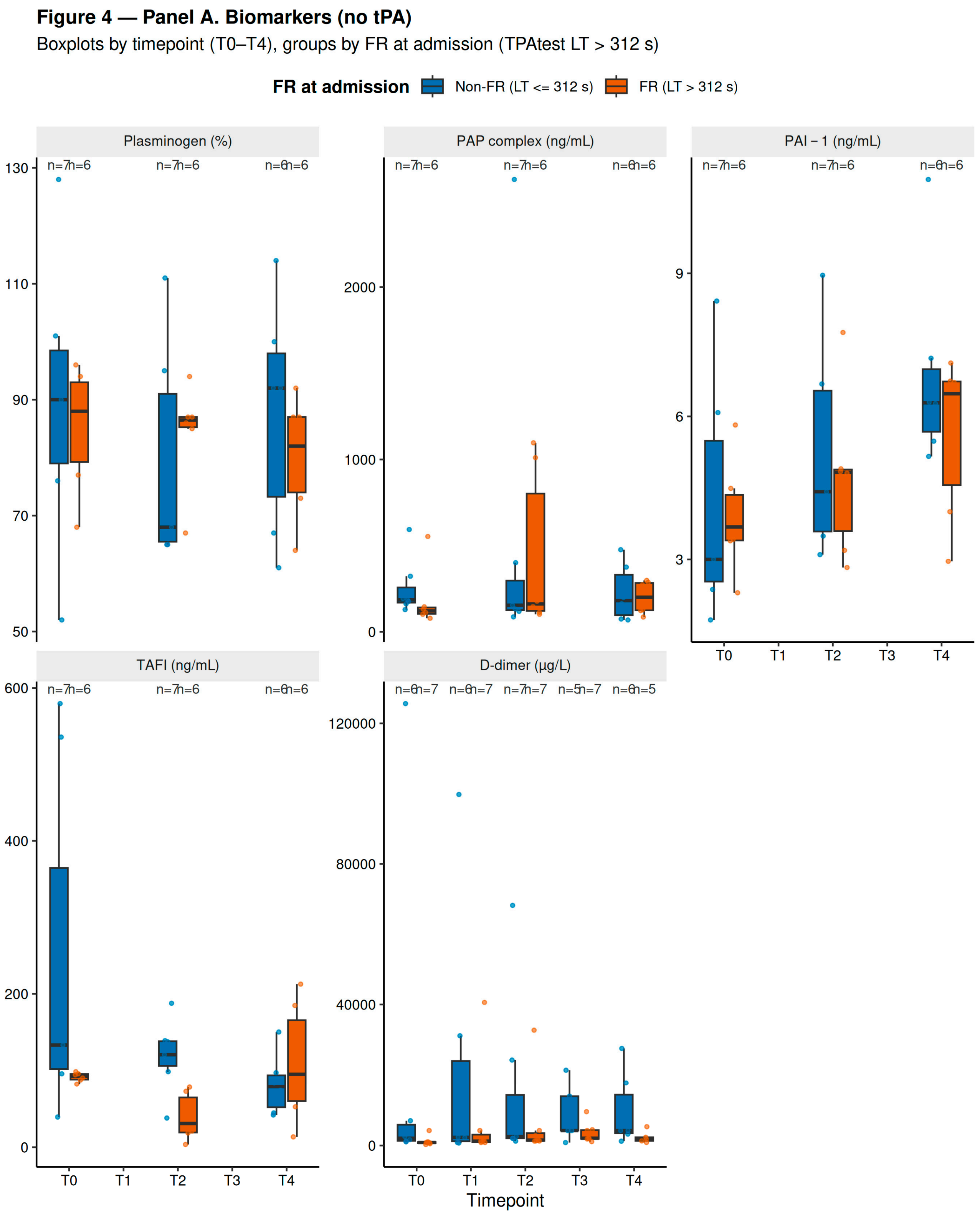

Figure S4: Boxplots display the temporal evolution of key fibrinolysis-related and thrombin-regulatory biomarkers—including plasminogen, plasmin–antiplasmin (PAP) complexes, plasminogen activator inhibitor-1 (PAI-1), thrombin-activatable fibrinolysis inhibitor (TAFI), and D-dimer—across T0–T4. Patients were stratified by fibrinolytic resistance (FR) at admission, defined as a TPA-test lysis time > 312 seconds. Non-FR patients (blue; LT ≤ 312 s) and FR patients (orange; LT > 312 s) are shown separately. The number of observations available at each timepoint and for each group is displayed above the panels. Boxplots represent medians and interquartile ranges with individual values overlaid. The figure highlights dynamic changes in antifibrinolytic capacity, plasmin generation, and fibrin turnover in FR versus non-FR phenotypes during the first 7 days of critical illness.

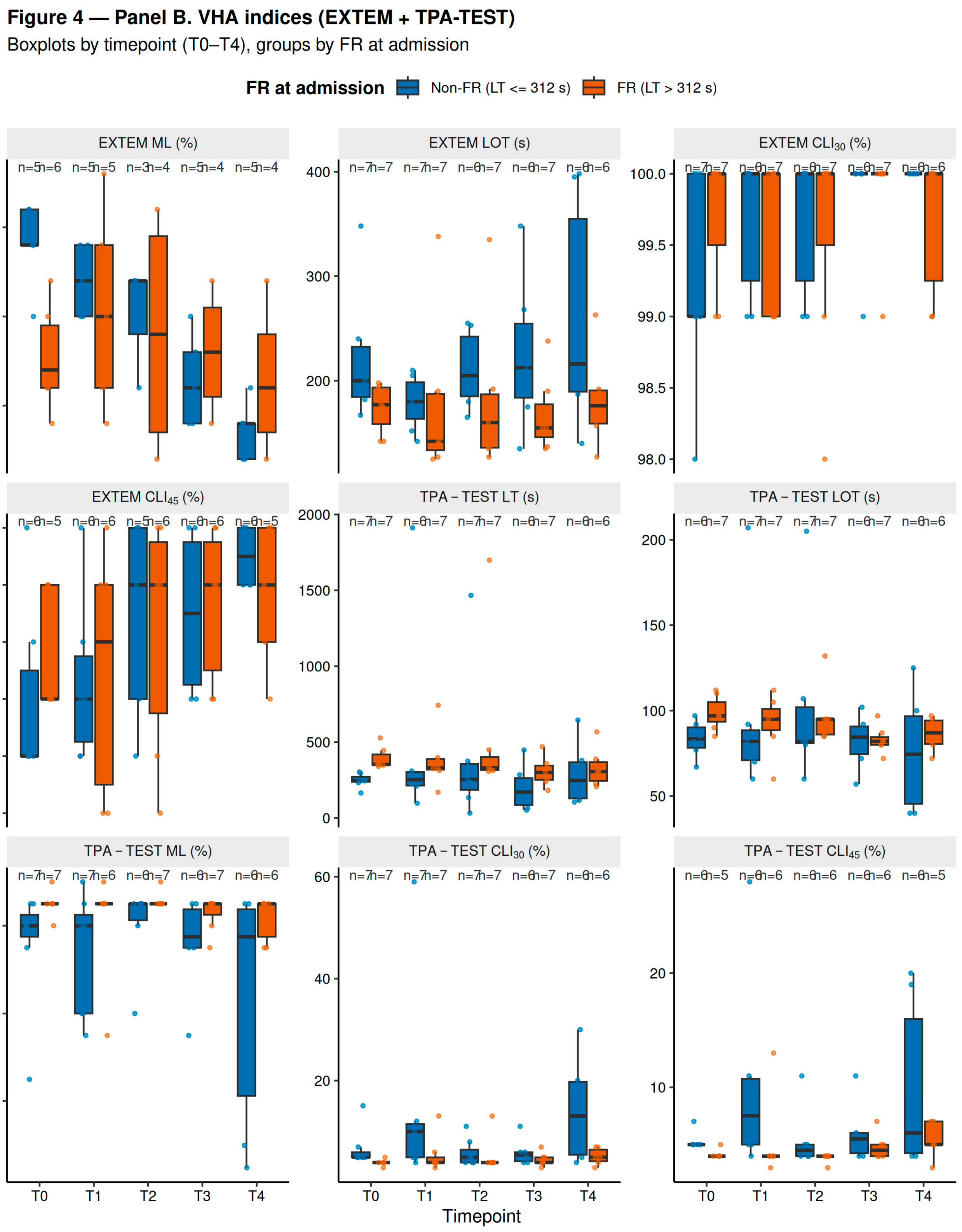

Figure S5: Boxplots illustrate the temporal evolution of viscoelastic parameters related to fibrinolysis and clot stability, including EXTEM maximum lysis (ML), lysis onset time (LOT), clot lysis at 30 minutes (CLI_{30}), clot lysis at 45 minutes (CLI_{45}), and corresponding TPA-test measures (LT, LOT, ML, CLI_{30}, CLI_{45}) across timepoints T0–T4. Patients were stratified by fibrinolytic resistance (FR) at admission, defined as a TPA-test lysis time > 312 seconds. Non-FR patients (blue; LT $\leq$ 312 s) and FR patients (orange; LT > 312 s) are shown separately. The number of valid observations at each timepoint is displayed above the panels. Boxplots depict medians and interquartile ranges, with individual values overlaid.

Supplementary — Complete VHA panel (incl. FIBTEM) — linear; n under ticks

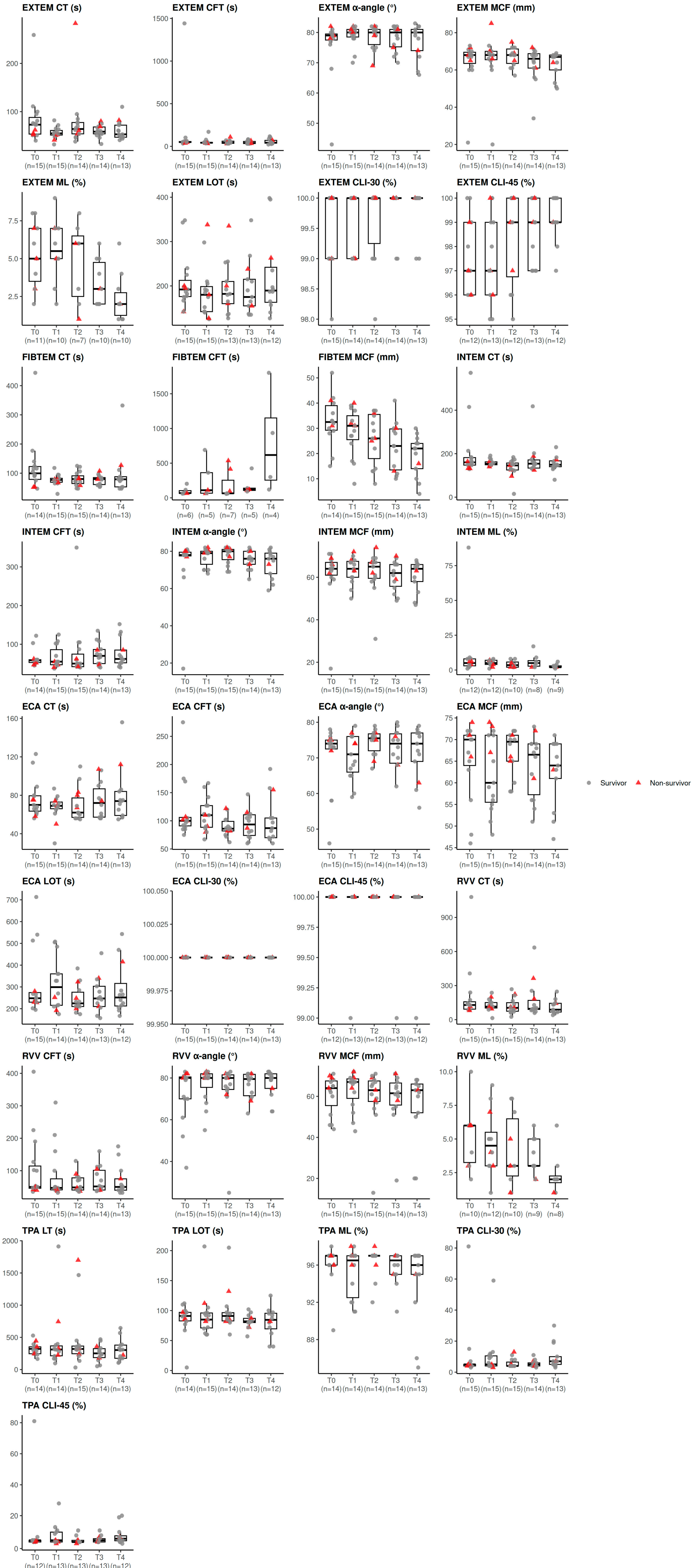

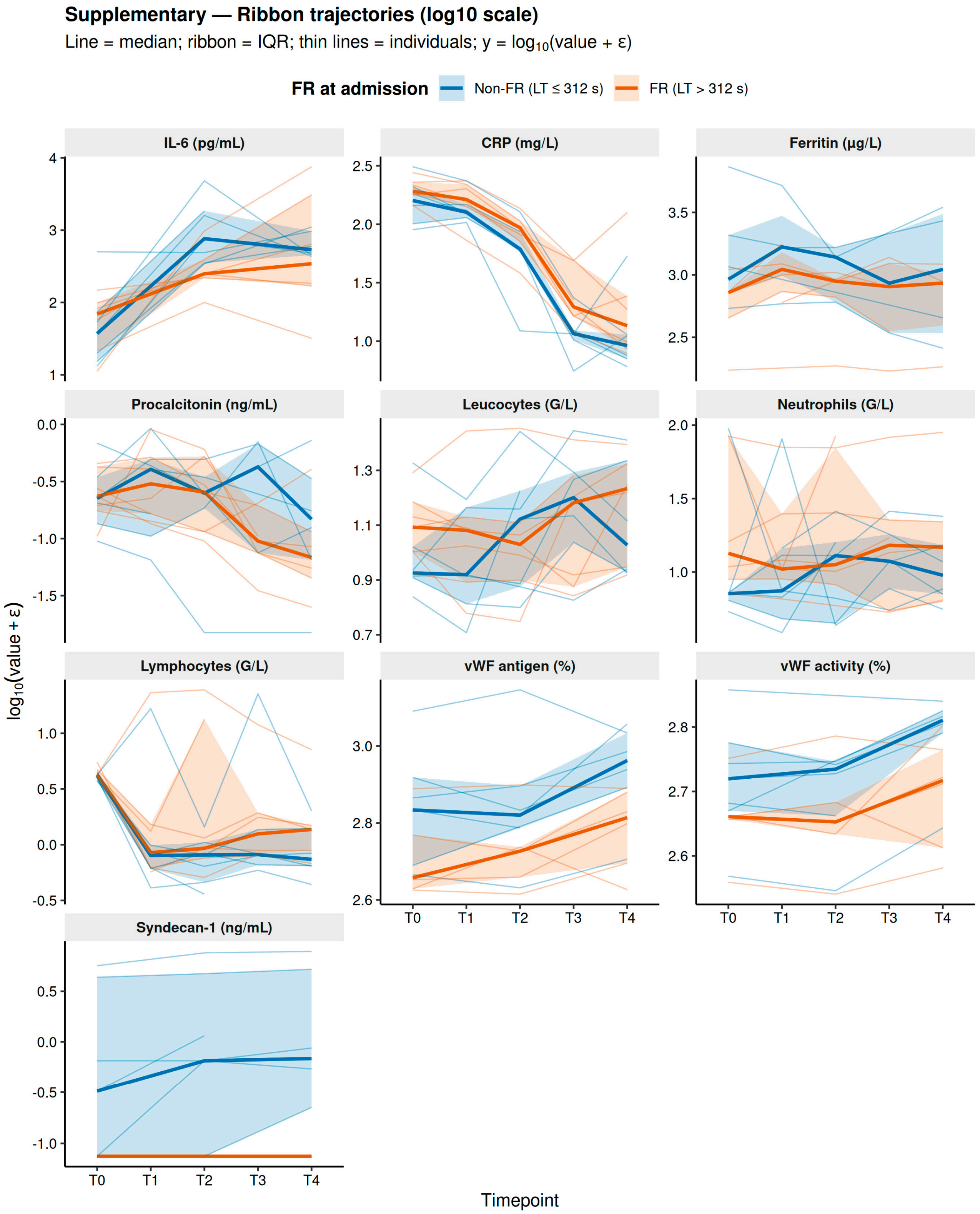

Figure S7: Ribbon plots depict the temporal evolution of key inflammatory (IL-6, CRP, Ferritin, Procalcitonin, Leukocytes, Neutrophils, Lymphocytes) and endothelial injury biomarkers (vWF antigen, vWF activity, Syndecan-1) across timepoints T0–T4. Median trajectories are shown as solid lines, with shaded ribbons denoting the interquartile range (IQR). Thin lines represent individual patient trajectories. Values were transformed using a log_{10}(value + ε) scaling to account for skewed distributions and zero-inflation. Patients were stratified by fibrinolytic resistance (FR) at admission, defined as TPA-test lysis time (LT) > 312 s. Non-FR patients (blue; LT ≤ 312 s) and FR patients (orange; LT > 312 s) are displayed separately.

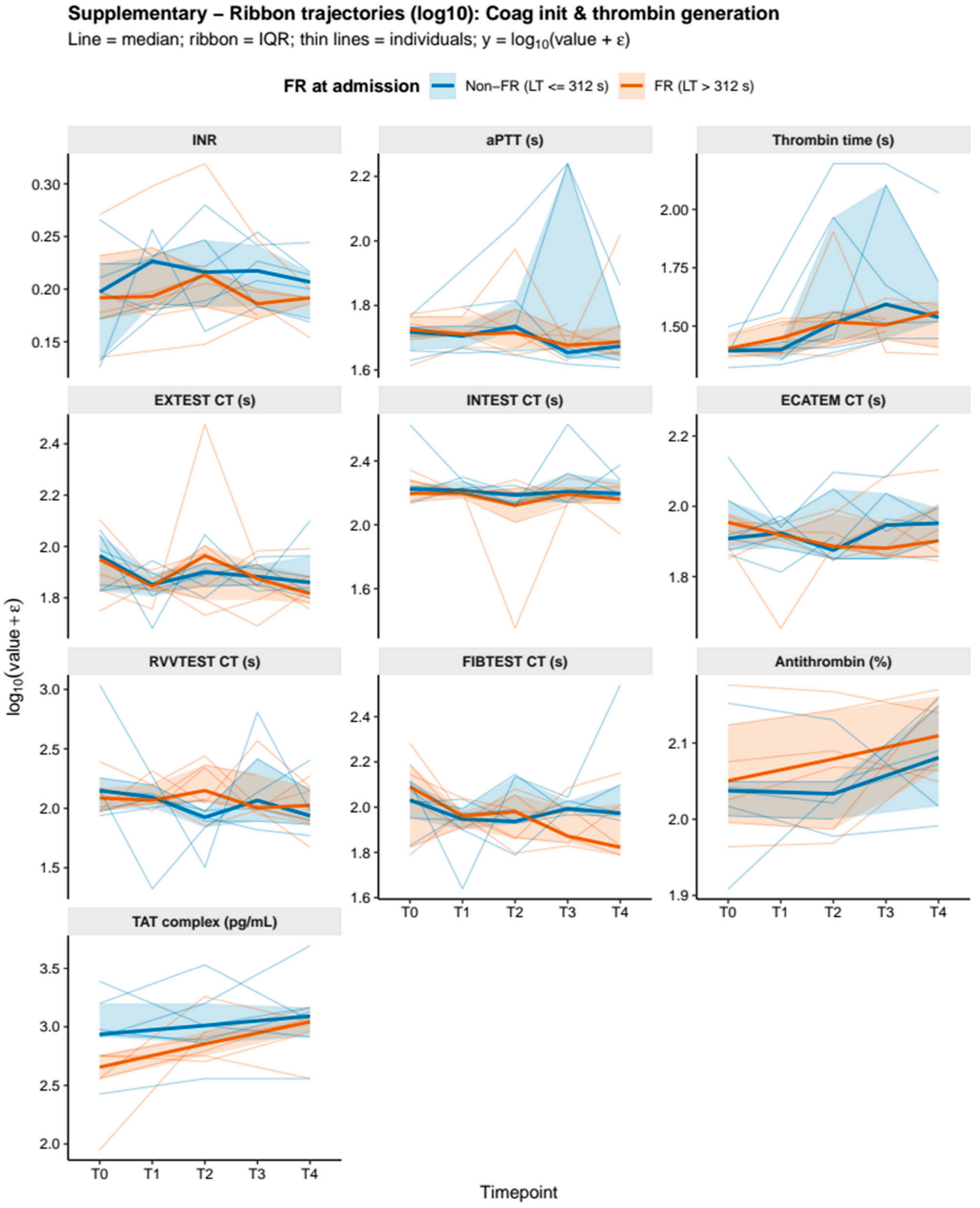

Figure S8: Ribbon plots display the temporal evolution of coagulation initiation parameters (INR, aPTT, Thrombin time), viscoelastic clotting times (EX-test CT, IN-test CT, ECA-test CT, RVV-test CT, FIB-test CT), and markers of thrombin generation (TAT complex, Antithrombin III) across timepoints T0–T4. Median values are represented by solid lines, shaded ribbons denote the interquartile range (IQR), and individual patient trajectories are shown as thin lines. All biomarkers were transformed using a log_{10}(value + ε) function to address right-skewed distributions and zero-inflation. Patients were stratified by fibrinolytic resistance (FR) at admission, defined as TPA-test lysis time > 312 seconds. Non-FR patients (blue) and FR patients (orange) are shown separately.

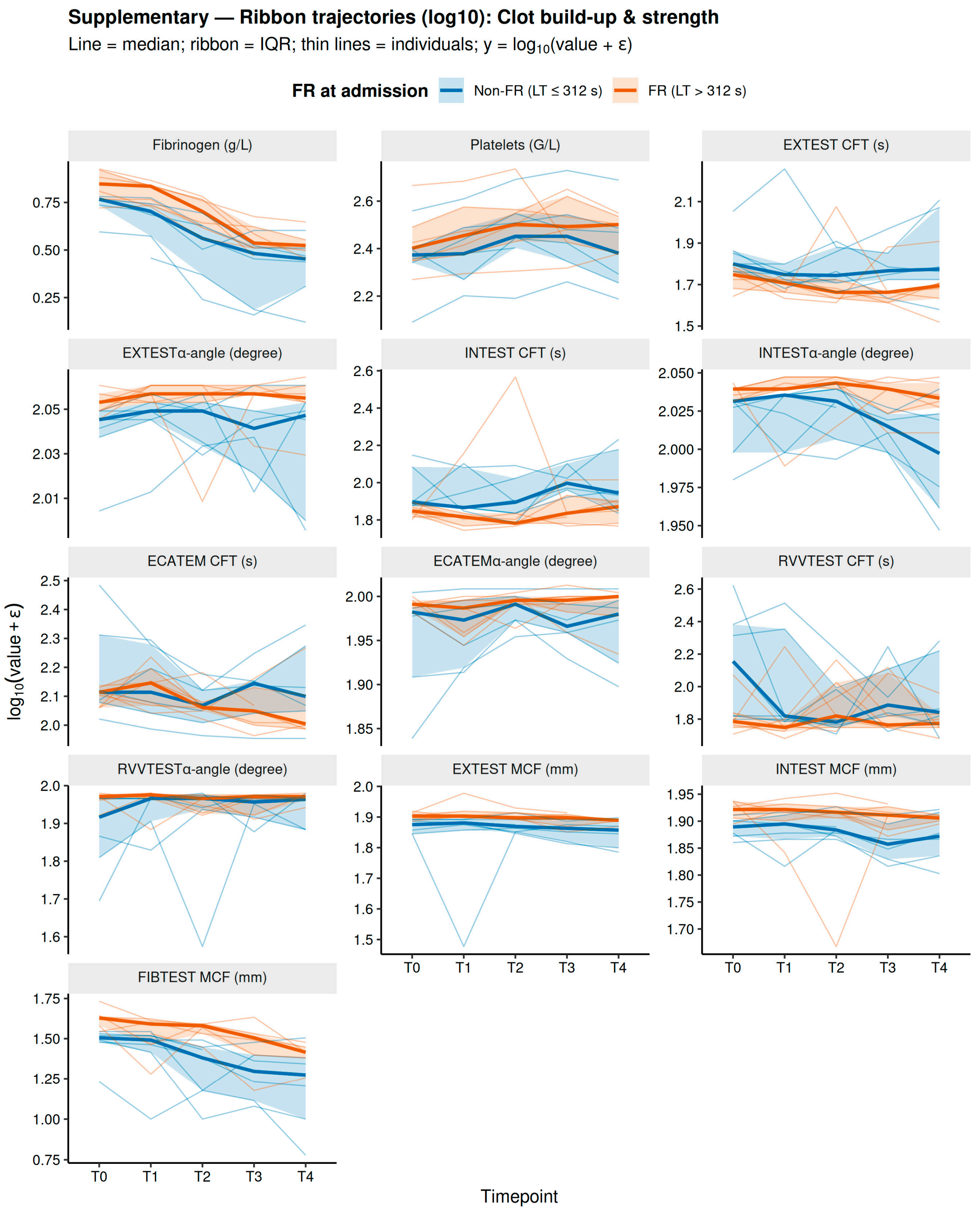

Figure S9: Ribbon plots display the temporal trajectories of clot formation and mechanical strength parameters across T0–T4, including fibrinogen concentration, platelet count, clot formation times (CFT) from EX-test, IN-test, ECATEM, and RVV-test, α-angles from EX-test, IN-test, ECATEM, and RVV-test, and maximum clot firmness (MCF) from EX-test, IN-test, and FIB-test. Median values are depicted by solid lines, shaded ribbons represent the interquartile range (IQR), and thin lines correspond to individual patient trajectories. All markers were transformed using a $\log_{10}$(value + ε) function to accommodate skewed distributions and zero values. Patients were stratified by fibrinolytic resistance (FR) at admission, defined as TPA-test lysis time > 312 seconds. Non-FR patients (blue) and FR patients (orange) are shown separately.

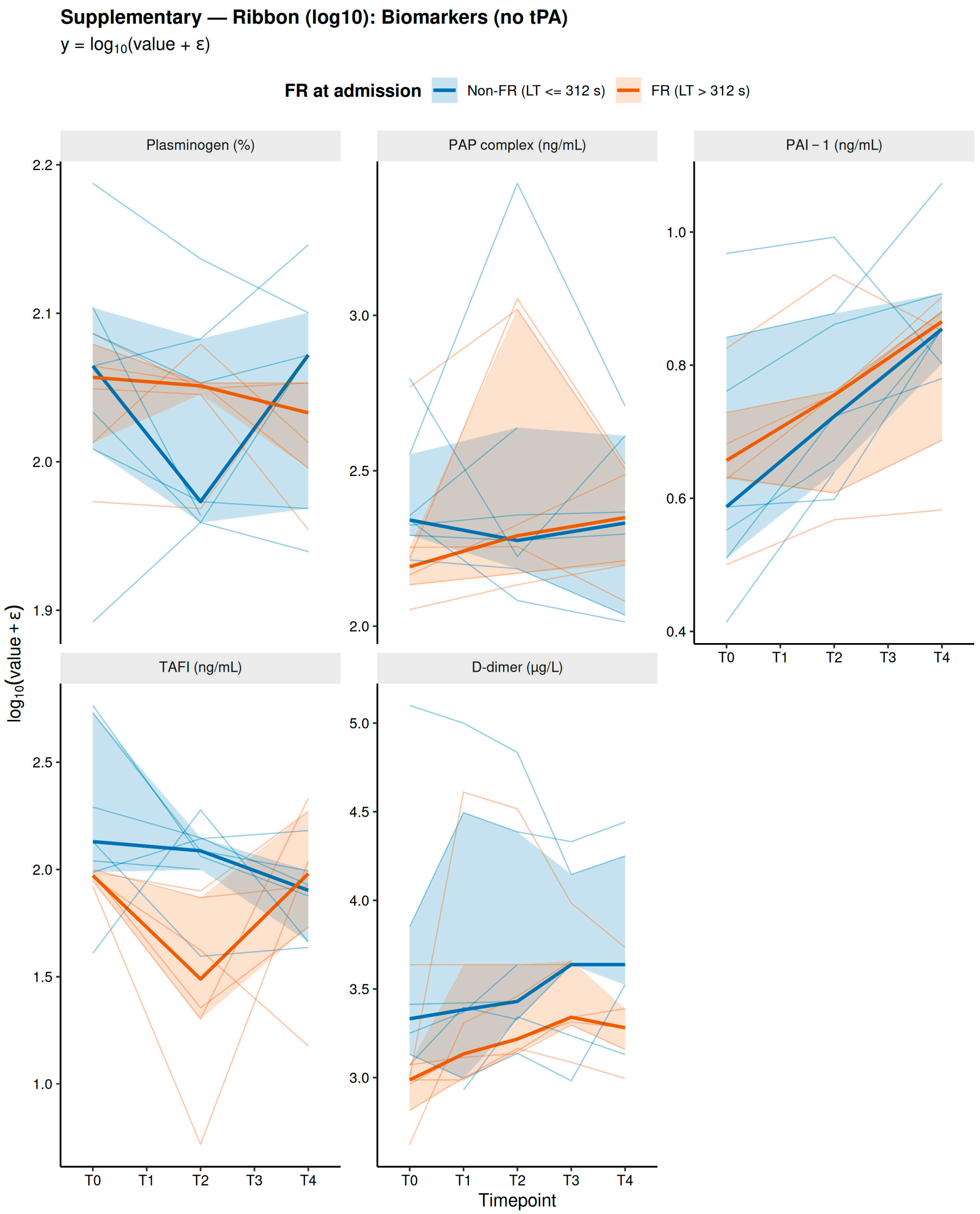

Figure S10: Ribbon plots illustrate the temporal evolution of plasminogen, plasmin–antiplasmin (PAP) complexes, plasminogen activator inhibitor-1 (PAI-1), thrombin-activatable fibrinolysis inhibitor (TAFI), and D-dimer from T0 to T4. Median trajectories are shown as solid lines, shaded ribbons represent the interquartile range (IQR), and thin lines depict individual patient trajectories. All biomarkers were transformed using $\log_{10}(\text{value} + \varepsilon)$ to address skewness and zero-inflation. Patients were stratified by fibrinolytic resistance (FR) at admission, defined as a TPA-test lysis time > 312 seconds. Non-FR patients (blue; LT ≤ 312 s) and FR patients (orange; LT > 312 s) are displayed separately.

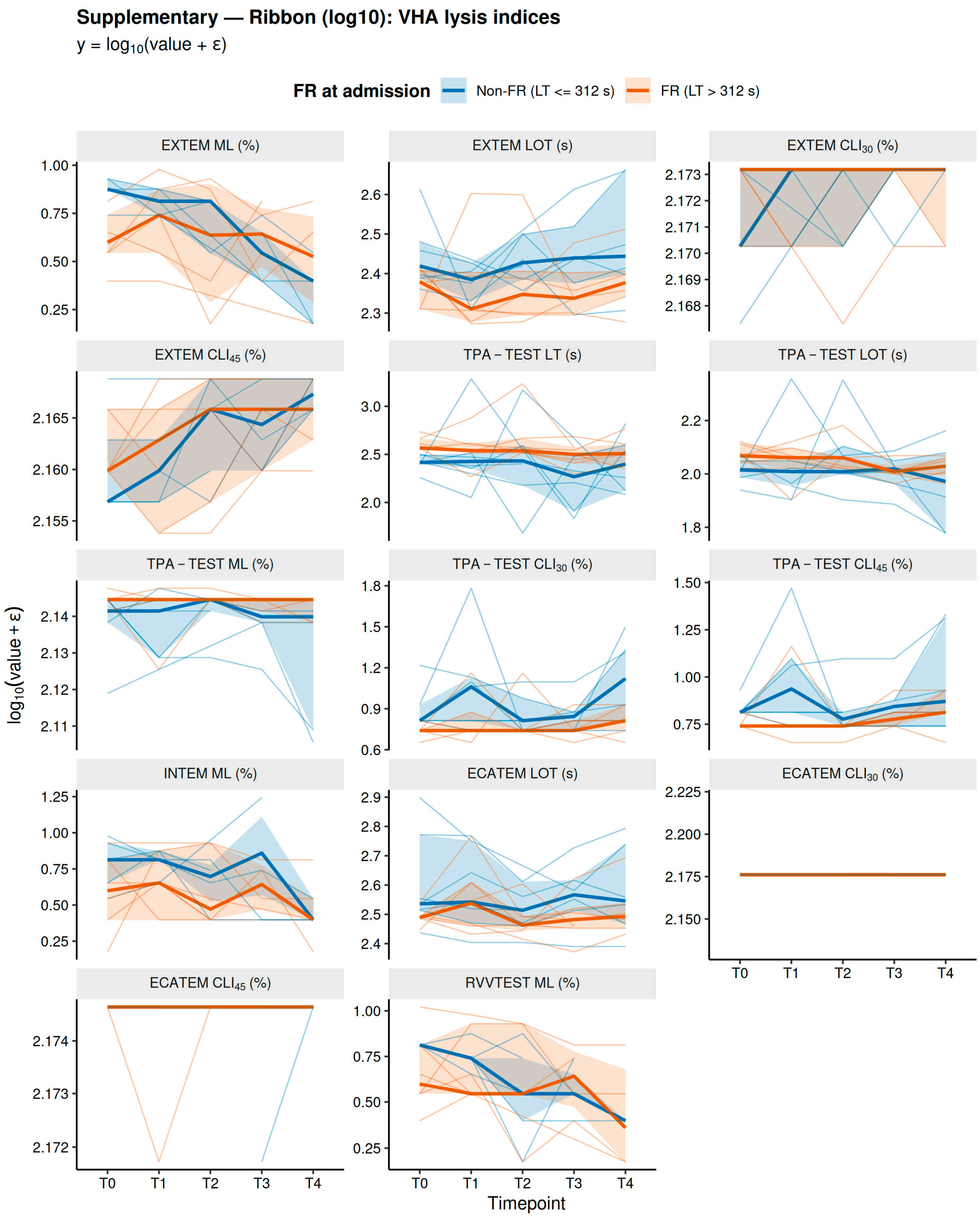

Figure S11: Ribbon plots depict temporal changes in clot lysis parameters obtained from EXTEM, TPA-test, INTEM, ECATEM, and RVV-test assays from T0 to T4. Parameters include maximum lysis (ML), lysis onset time (LOT), clot lysis at 30 minutes (CLI_{30}), and clot lysis at 45 minutes (CLI_{45}). Median trajectories are shown as solid lines, interquartile ranges (IQRs) as shaded ribbons, and thin lines represent individual patient trajectories. All indices were transformed using $\log_{10}(\text{value} + \varepsilon)$ to mitigate skewness and zero inflation. Patients were stratified by fibrinolytic resistance (FR) at admission, defined as TPA-test lysis time > 312 seconds. Non-FR patients (blue; LT ≤ 312 s) and FR patients (orange; LT > 312 s) are shown separately.

Table S3: Trajectories of inflammation and hemostasis parameters across time points (T0-T4)

**marker**	**T0**	**T1**	**T2**	**T3**	T4
IL-6 (pg/mL)	49.63 [16.18–79.16]		389.70 [246.00–975.00]		535.65 [367.23–999.45]
CRP (mg/L)	180.90 [150.75–211.00]	139.40 [117.30–180.30]	69.50 [57.90–91.75]	12.50 [9.72–19.90]	8.00 [5.70–16.80]
Ferritin (µg/L)	673.00 [602.75–1105.25]	1168.00 [811.00–1618.00]	864.00 [606.00–1123.00]	836.00 [293.50–1215.50]	826.00 [396.00–1161.00]
Procalcitonin (ng/mL)	0.22 [0.18–0.34]	0.39 [0.16–0.47]	0.24 [0.13–0.33]	0.10 [0.07–0.20]	0.11 [0.05–0.17]
Leucocytes (G/L)	8.40 [6.70–12.71]	8.88 [6.30–12.32]	9.88 [6.12–13.91]	13.89 [7.24–17.81]	14.86 [7.00–19.40]
vWF antigen (%)	449.30 [321.60–595.30]		474.80 [395.20–543.40]		629.95 [460.77–756.12]
vWF activity (%)	351.70 [341.80–438.00]		354.85 [328.67–437.45]		483.95 [380.73–526.58]
Syndecan-1 (ng/mL)	0.00 [0.00–0.25]		0.00 [0.00–0.57]		0.00 [0.00–0.55]
aPTT (s)	40.40 [36.00–44.70]	37.85 [36.92–43.78]	39.70 [38.32–46.57]	33.80 [30.85–38.73]	35.45 [30.78–42.70]
Thrombin time (s)	18.10 [17.40–21.60]	19.20 [17.20–24.55]	26.40 [20.03–29.43]	25.30 [20.77–33.98]	28.40 [22.25–32.65]
EXTEM CT (s)	73.00 [53.50–88.00]	55.00 [51.50–61.00]	64.00 [54.00–77.50]	58.50 [54.00–68.25]	53.00 [47.00–72.00]
INTEM CT (s)	160.00 [147.50–182.00]	154.00 [149.00–162.50]	146.00 [125.00–157.00]	154.00 [133.75–171.25]	149.00 [140.00–167.00]
Antithrombin (%)	82.00 [74.00–92.00]		82.00 [73.00–96.00]		94.50 [88.00–114.75]
TAT complex (pg/mL)	476.82 [272.13–773.34]		729.39 [533.19–935.79]		1028.87 [794.21–1273.95]
Fibrinogen (g/L)	5.46 [5.10–6.45]	4.98 [4.50–5.95]	3.80 [2.98–4.55]	2.80 [2.42–3.30]	2.60 [2.12–2.96]
Platelets (G/L)	195.00 [180.00–218.00]	219.00 [178.50–262.00]	270.00 [220.00–312.50]	261.00 [191.75–306.25]	253.00 [164.00–277.00]
EXTEM CFT (s)	50.00 [46.00–56.00]	42.00 [38.50–46.00]	41.00 [35.00–60.50]	38.50 [32.75–57.50]	42.00 [37.00–70.00]
EXTEM α-angle (°)	79.00 [77.50–79.50]	80.00 [78.50–81.00]	80.00 [76.00–81.00]	80.00 [75.25–81.00]	80.00 [74.00–81.00]
FIBTEM MCF (mm)	32.50 [29.25–39.00]	31.00 [25.50–35.00]	26.00 [18.00–35.50]	23.00 [13.50–29.75]	22.00 [14.00–24.00]
EXTEM MCF (mm)	68.00 [63.50–69.50]	68.00 [65.50–70.00]	68.00 [63.50–71.25]	66.00 [61.00–68.75]	67.00 [60.00–68.00]
Plasminogen (%)	90.00 [77.00–96.00]		86.00 [67.00–87.00]		87.00 [71.50–92.00]
PAP complex (ng/mL)	161.80 [128.95–192.70]		154.25 [118.70–401.00]		181.00 [113.65–290.25]
PAI-1 (ng/mL)	3.42 [2.70–4.90]		4.81 [3.49–6.40]		6.29 [5.40–6.83]
α2-Antiplasmin (%)	110.00 [107.00–116.00]		101.00 [88.00–109.00]		100.00 [90.75–106.00]
TAFI (ng/mL)	95.40 [89.90–133.05]		77.65 [37.65–120.40]		82.40 [50.16–118.41]
D-dimer (µg/L)	1125.50 [837.50–2347.50]	2045.00 [930.25–5925.00]	2550.00 [1415.00–4200.00]	4200.00 [1920.00–4420.00]	3540.00 [1652.25–4470.00]
EXTEM ML (%)	5.00 [3.50–7.00]	5.50 [5.00–7.00]	6.00 [2.50–6.50]	3.00 [2.00–4.75]	2.00 [1.25–2.75]
EXTEM LOT (s)	192.00 [176.00–212.50]	180.00 [142.00–198.50]	182.00 [160.00–210.00]	175.00 [155.00–215.00]	189.50 [163.00–242.00]
EXTEM CLI-30 (%)	100.00 [99.00–100.00]	100.00 [99.00–100.00]	100.00 [99.25–100.00]	100.00 [100.00–100.00]	100.00 [100.00–100.00]
EXTEM CLI-45 (%)	97.00 [96.00–99.00]	97.00 [96.00–99.00]	99.00 [96.75–100.00]	99.00 [97.00–100.00]	99.00 [99.00–100.00]
ECATEM LOT (s)	248.00 [230.00–274.00]	299.00 [215.50–360.00]	224.50 [210.00–275.75]	247.00 [210.00–303.00]	251.50 [212.50–316.00]
ECATEM CLI-30 (%)	100.00 [100.00–100.00]	100.00 [100.00–100.00]	100.00 [100.00–100.00]	100.00 [100.00–100.00]	100.00 [100.00–100.00]
ECATEM CLI-45 (%)	100.00 [100.00–100.00]	100.00 [100.00–100.00]	100.00 [100.00–100.00]	100.00 [100.00–100.00]	100.00 [100.00–100.00]
TPA LT (s)	321.50 [245.75–353.00]	311.50 [213.75–367.50]	320.00 [246.00–365.00]	251.50 [185.75–324.75]	303.00 [175.00–380.00]
TPA LOT (s)	91.00 [82.75–97.00]	85.00 [71.00–96.00]	91.00 [82.75–96.50]	82.00 [80.00–87.00]	84.50 [69.50–95.50]
TPA ML (%)	97.00 [96.00–97.00]	96.50 [92.50–97.00]	97.00 [97.00–97.00]	96.50 [95.00–97.00]	96.00 [95.00–97.00]
TPA CLI-30 (%)	5.00 [4.00–5.00]	5.00 [4.00–10.50]	4.00 [4.00–6.50]	5.00 [4.00–6.00]	7.00 [5.00–10.00]
TPA CLI-45 (%)	5.00 [4.00–5.00]	5.00 [4.00–10.00]	4.00 [4.00–5.00]	5.00 [4.00–6.00]	6.00 [4.75–7.75]

Table S4. ClotPro viscoelastic assays and parameters: physiological basis and interpretive framework

Assay	Parameter	Physiological process modeled	What the parameter represents	General interpretive meaning
**EX-test** (tissue factor activation)	**CT**	Initiation of coagulation (extrinsic + common pathway)	Time from test start to initial clot formation (2 mm amplitude)	Prolongation reflects delayed thrombin generation due to coagulation factor deficiency or anticoagulant effect
	**CFT**	Clot propagation kinetics	Time required for clot growth from 2 to 20 mm amplitude	Prolongation suggests impaired fibrin polymerization and/or platelet contribution
	**α-angle**	Rate of fibrin network formation	Speed of clot build-up	Reduced angle indicates slower clot propagation
	**MCF**	Clot mechanical strength	Maximum clot firmness during measurement	Reflects combined fibrinogen concentration and platelet contribution
	**ML**	Net clot breakdown	Maximum reduction in clot firmness (% of MCF)	Reduced values indicate limited net clot breakdown, influenced by fibrinolysis and clot contraction
	**CLI-30 / CLI-45**	Clot stability over time	Residual clot firmness at 30 or 45 minutes	Higher values indicate increased resistance to spontaneous clot breakdown
**FIB-test** (platelet inhibition)	**CT**	Initiation of fibrin-based clot formation	Time to clot initiation with platelet contribution suppressed	Prolongation suggests reduced coagulation factor activity
	**MCF**	Fibrin-based clot strength	Maximum firmness attributable primarily to fibrinogen	Reduced values reflect impaired fibrinogen quantity or function
**IN-test** (contact activation)	**CT**	Initiation of coagulation (intrinsic + common pathway)	Time to clot initiation following surface-dependent activation	Prolongation suggests intrinsic pathway factor deficiency
	**CFT / α-angle**	Clot propagation kinetics	Speed and efficiency of clot formation	Alterations reflect impaired propagation dynamics
	**MCF**	Clot mechanical strength	Maximum firmness of the clot	Reflects global clot stability
**HI-test** (heparinase-modified IN-test)	**CT**	Intrinsic pathway without heparin effect	Clot initiation after heparin neutralization	Discrepancy vs IN-test suggests heparin effect
**TPA-test** (exogenous tPA challenge)	**LOT**	Initiation of fibrinolysis	Delay between clot formation and onset of lysis	Prolongation indicates delayed activation of fibrinolysis
	**LT**	Fibrinolytic responsiveness	Time from clot formation to 50% clot breakdown under standardized tPA stimulation	Prolongation reflects relative fibrinolytic resistance
	**ML**	Extent of clot breakdown under challenge	Maximum lysis during measurement	Reduced values indicate impaired fibrinolytic response
**RVV-test** (FX activation by Russell viper venom)	**CT**	Common pathway activation from factor X	Time to clot initiation independent of upstream pathways	Prolongation reflects FX-directed anticoagulant effects
	**MCF**	Clot mechanical strength	Maximum firmness of the clot	Reflects downstream clot stability
**ECA-test** (direct prothrombin activation)	**CT**	Thrombin generation	Time to clot initiation via direct prothrombin activation	Prolongation reflects thrombin inhibition
	**LOT / LT**	Fibrinolysis timing	Onset and progression of clot breakdown	Alterations reflect fibrinolytic dynamics under thrombin-driven clot formation

Table S5 Normal values on ClotPro as indexed by manufacturer.

Test	CT (s)	CFT (s)	A5 (mm)	A10 (mm)	A20 (mm)	MCF (mm)	ML (%)	LT (s)
**EX**	38–65	42–93	39–58	47–64	52–67	53–68	–	–
**FIB**	–	–	6–21	7–23	8–25	9–27	–	–
**AP**	44–76	46–118	36–59	46–65	51–67	51–67	–	–
**IN**	139–187	52–139	32–53	41–61	48–65	49–65	–	–
**HI**	141–185	47–108	36–56	45–61	49–65	49–65	–	–
**TPA**	29–58	–	–	–	–	20–44	88–96	151–411
**RVV**	48–77	–	–	47–63	53–67	54–68	–	–
**ECA**	68–100	–	–	54–66	58–70	61–72	–	–

Supplementary Methods S2. Laboratory Assays and ELISA Characteristics

Concentrations of Human thrombin activable fibrinolysis inhibitor (TAFI): detection range: 2.5-160 ng/mL, Human thrombin antitrombin complex (TAC): detection range: 31.2-1000 pg/mL, Human plasmin/antiplasmin complex (PAP): detection range: 0,78-50 ng/mL, Human Syndecan 1(SDC1): detection range: 1.56-100 ng/mL, Human plasminogen activator inhibitor (PAI): detection range: 0.312-20 ng/mL, Human tissue plasminogen activator (tPA): detection range: 0.156-10 ng/mL were measured using enzyme-linked immunosorbent assay (ELISA) kits from MyBioSource, San Diego, CA, USA) in the Szentágothai Research Center (University of Pécs) following the manufacturer's protocol.

References:

1. Coupland, L.A.; Rabbolini, D.J.; Schoenecker, J.G.; Crispin, P.J.; Miller, J.J.; Ghent, T.; Medcalf, R.L.; Aneman, A.E. Point-of-Care Diagnosis and Monitoring of Fibrinolysis Resistance in the Critically Ill: Results from a Feasibility Study. Crit. Care 2023, 27(1), 55. doi:10.1186/s13054-023-04329-5

Table S. STROBE Statement—checklist of items that should be included in reports of observational studies

	Item No	Recommendation	Page No
**Title and abstract**	1	(*a*) Indicate the study's design with a commonly used term in the title or the abstract	1
		(*b*) Provide in the abstract an informative and balanced summary of what was done and what was found	2
**Introduction**			
Background/rationale	2	Explain the scientific background and rationale for the investigation being reported	2-3
Objectives	3	State specific objectives, including any prespecified hypotheses	3
**Methods**			
Study design	4	Present key elements of study design early in the paper	4
Setting	5	Describe the setting, locations, and relevant dates, including periods of recruitment, exposure, follow-up, and data collection	4
Participants	6	(*a*) *Cohort study*—Give the eligibility criteria, and the sources and methods of selection of participants. Describe methods of follow-up<br>*Case-control study*—Give the eligibility criteria, and the sources and methods of case ascertainment and control selection. Give the rationale for the choice of cases and controls<br>*Cross-sectional study*—Give the eligibility criteria, and the sources and methods of selection of participants	4
		(*b*) *Cohort study*—For matched studies, give matching criteria and number of exposed and unexposed<br>*Case-control study*—For matched studies, give matching criteria and the number of controls per case	NA
Variables	7	Clearly define all outcomes, exposures, predictors, potential confounders, and effect modifiers. Give diagnostic criteria, if applicable	4-5
Data sources/ measurement	8*	For each variable of interest, give sources of data and details of methods of assessment (measurement). Describe comparability of assessment methods if there is more than one group	*4-5*
Bias	9	Describe any efforts to address potential sources of bias	5
Study size	10	Explain how the study size was arrived at	5
Quantitative variables	11	Explain how quantitative variables were handled in the analyses. If applicable, describe which groupings were chosen and why	5
Statistical methods	12	(*a*) Describe all statistical methods, including those used to control for confounding	5-6
		(*b*) Describe any methods used to examine subgroups and interactions	5-6
		(*c*) Explain how missing data were addressed	5-6

	(*d*) *Cohort study*—If applicable, explain how loss to follow-up was addressed<br>*Case-control study*—If applicable, explain how matching of cases and controls was addressed<br>*Cross-sectional study*—If applicable, describe analytical methods taking account of sampling strategy	6
	(*e*) Describe any sensitivity analyses	NA

Results

Participants	13 *	(a) Report numbers of individuals at each stage of study—eg numbers potentially eligible, examined for eligibility, confirmed eligible, included in the study, completing follow-up, and analysed	6
		(b) Give reasons for non-participation at each stage	NA
		(c) Consider use of a flow diagram	NA
Descriptive data	14 *	(a) Give characteristics of study participants (eg demographic, clinical, social) and information on exposures and potential confounders	6-8
		(b) Indicate number of participants with missing data for each variable of interest	Suppl 1-3
		(c) *Cohort study*—Summarise follow-up time (eg, average and total amount)	NA
Outcome data	15 *	*Cohort study*—Report numbers of outcome events or summary measures over time	*8-13*
		Case-control study—Report numbers in each exposure category, or summary measures of exposure	
		Cross-sectional study—Report numbers of outcome events or summary measures	
Main results	16	(*a*) Give unadjusted estimates and, if applicable, confounder-adjusted estimates and their precision (eg, 95% confidence interval). Make clear which confounders were adjusted for and why they were included	8-13
		(*b*) Report category boundaries when continuous variables were categorized	5
		(*c*) If relevant, consider translating estimates of relative risk into absolute risk for a meaningful time period	NA
Other analyses	17	Report other analyses done—eg analyses of subgroups and interactions, and sensitivity analyses	13-14

Discussion

Key results	18	Summarise key results with reference to study objectives	14
Limitations	19	Discuss limitations of the study, taking into account sources of potential bias or imprecision. Discuss both direction and magnitude of any potential bias	16
Interpretation	20	Give a cautious overall interpretation of results considering objectives, limitations, multiplicity of analyses, results from similar studies, and other relevant evidence	16
Generalisability	21	Discuss the generalisability (external validity) of the study results	16

Other information

Funding	22	Give the source of funding and the role of the funders for the present study and, if applicable, for the original study on which the present article is based	18

*Give information separately for cases and controls in case-control studies and, if applicable, for exposed and unexposed groups in cohort and cross-sectional studies.

Note: An Explanation and Elaboration article discusses each checklist item and gives methodological background and published examples of transparent reporting. The STROBE checklist is best used in conjunction with this article (freely available on the Web sites of PLoS Medicine at http://www.plosmedicine.org/, Annals of Internal Medicine at http://www.annals.org/, and Epidemiology at http://www.epidem.com/). Information on the STROBE Initiative is available at www.strobe-statement.org.
